# NADPH oxidase 2 (NOX2)-independent inducers of neutrophil extracellular traps promote resolution of chronic inflammation

**DOI:** 10.1016/j.redox.2026.104302

**Published:** 2026-07-15

**Authors:** M. Euler, O. Hattab, K. Borah Slater, B. Golub, R. Selin, Z. Cheng, Y.A. Maluje Villanueva, G.H. Özkan, G. Bila, K. Dutta, D. Weidner, E.J. Hoffmann, J. Friscic, C.D. Sadik, T. Harrer, J. Köhl, G. Schett, L. Munoz, A. Fähnrich, S. Murthy, R. Grieshaber-Bouyer, M. Herrmann, R. Bilyy, H.R. Griffiths, A. Mokhir, M.H. Hoffmann

**Affiliations:** a Department of Internal Medicine 3 – Rheumatology and Immunology, Friedrich-Alexander-Universität Erlangen-Nürnberg and Universitätsklinikum Erlangen, Erlangen, Germany; b Deutsches Zentrum Immuntherapie (DZI), Friedrich-Alexander-Universität Erlangen-Nürnberg and Universitätsklinikum Erlangen, Erlangen, Germany; c Biosciences, Faculty of Health and Life Sciences, University of Exeter, EX4 4QD, United Kingdom; d Friedrich-Alexander University Erlangen-Nürnberg (FAU), Department of Chemistry and Pharmacy, Organic Chemistry Chair II, Erlangen, Germany; e Medical Systems Biology, Institute of Experimental Dermatology, University of Lübeck, Lübeck, 23562, Germany; f Institute of Cellular Biology and Pathology “Nicolae Simionescu”, Bucharest, Romania; g Department of Histology, Cytology and Embryology, Danylo Halytsky Lviv National Medical University, Lviv, 79010, Ukraine; h University of Luebeck, Department of Dermatology, Lübeck, 23562, Germany; i University of Luebeck, Institute for Systemic Inflammation Research, Lübeck, 23562, Germany; j Department of Rheumatology and Immunology, West China Hospital, Sichuan University, Chengdu, 610041, China; k Swansea University Medical School, Swansea University, Swansea, United Kingdom

**Keywords:** ROS, NETosis, NOX2, Inflammation, Arthritis, Prodrugs

## Abstract

Reactive oxygen species (ROS) generated by NADPH oxidase 2 (NOX2) are essential for antimicrobial defense but also for the resolution of inflammation. NOX2 deficiency, as observed in chronic granulomatous disease (CGD), predisposes to persistent sterile inflammation. Current therapeutic strategies largely rely on nonspecific immunosuppression or require residual NOX2 activity, while systemic ROS-inducing therapies are limited by toxicity.

Here, we present a NOX2-independent approach to restore inflammation-resolving ROS signaling using N-alkylaminoferrocene–based prodrugs (pro-NAAFs), which amplify pre-existing ROS rather than generating ROS indiscriminately. Among several candidates, prodrug 1 emerged as the most potent and well-tolerated ROS amplifier. In human neutrophils, prodrug 1 induced strong ROS production and neutrophil extracellular trap (NET) formation. These responses occurred independently of NOX2 and were maintained in CGD-derived neutrophils. Similar NOX2-independent ROS induction and NET formation were observed in immune cells from wild-type and NOX2-dysfunctional *Ncf1∗∗* mice. Prodrug 1-induced NETs aggregated into high-density structures (aggNETs) capable of degrading pro-inflammatory mediators *in vitro*. Prodrug 1 also inhibited neutrophil inflammasome activation. *In vivo*, subcutaneous administration of prodrug 1 reduced inflammatory mediator levels in air pouches, promoted resolution of chronic arthritis in *Ncf1∗∗* mice, and protected from bone destruction. Transcriptomic analyses indicated early suppression of inflammatory pathways and restoration of neutrophil maturation trajectories towards a wild-type–like state. While prodrug 1 increased protein oxidation markers, it shifted systemic oxysterol profiles towards an inflammation-resolving phenotype. These findings identify pro-NAAFs as NOX2-independent ROS amplifiers capable of restoring inflammatory resolution and highlight their therapeutic potential for chronic inflammatory conditions linked to NOX2-dysfunction.

## Highlights

• Pro-NAAFs induce aggregated NET (aggNET)-formation independently of NOX2 activity.
• The prototype pro-NAAF prodrug 1 restores ROS signaling in CGD-derived neutrophils.
• Prodrug 1-induced aggNETs degrade cytokines and promote resolution of inflammation.
• Prodrug 1 resolves chronic arthritis in NOX2-dysfunctional mice.
• Targeted ROS amplification by prodrug 1 limits systemic oxidative stress.

## Graphical abstract

**Figure:**
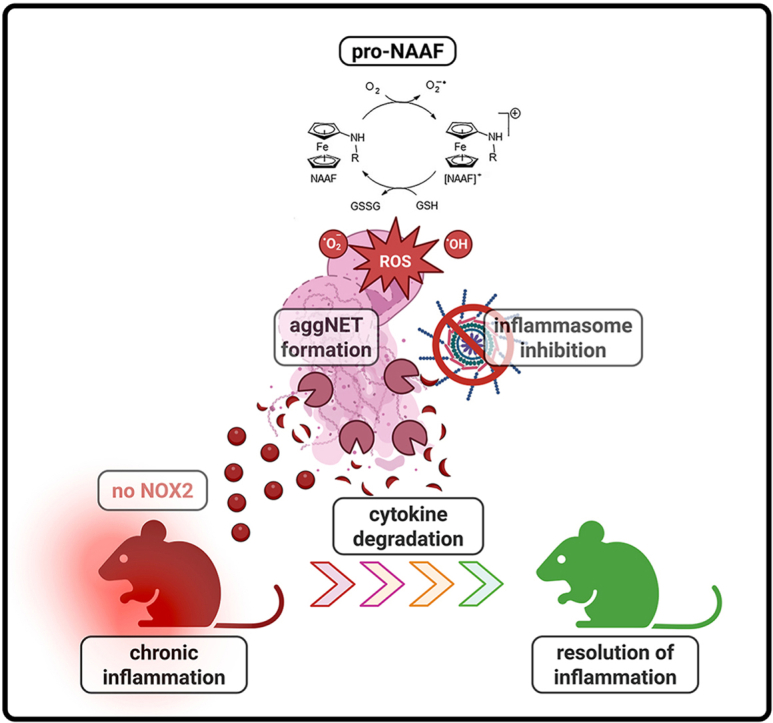

## 1 Introduction

Reactive oxygen species (ROS) include singlet oxygen (^1^O_2_), superoxide anion radicals (O_2_^•-^), hydrogen peroxide (H_2_O_2_), hydroxyl radicals (HO•), and hypochlorous acid (HOCl). In most biochemical reactions ROS act as strong oxidants, e.g., oxidizing thiols to disulfides [1], or inducing hydrogen subtraction from C–H bonds [2]. Polymorphonuclear neutrophils (PMNs) release ROS upon activation in an NADPH oxidase 2 (NOX2)-catalyzed reaction (oxidative burst) that initiates pathogen neutralization and destruction of sterile inflammatory irritants. Classically, ROS were considered pro-inflammatory agents that cause collateral damage to surrounding tissues. However, it is now also established that full (as in chronic granulomatous disease (CGD) [3,4]) or partial (as for example in arthritis [[5], [6], [7]], systemic lupus erythematosus (SLE) [8,9] and Chronic Inflammatory Demyelinating Polyneuropathy [10]) NOX2 deficiency results in chronic inflammation in humans and rodent models. Thus, ROS are required for the resolution of the residual inflammation once the pathogens or sterile triggers have been eradicated. Some of the possible mechanisms for the anti-inflammatory effect of ROS on innate immunity are its effects on phagocytosis [11] and the ROS-dependent generation of neutrophil extracellular traps (NETs) [[12], [13], [14]]. We have previously shown that high-density (aggregated) NETs (aggNETs) can limit innate immunity-driven inflammation in several organ systems by locally degrading cytokines and chemokines [8,12,13,15]. Thus, rather than acting in a binary pro-inflammatory or anti-inflammatory manner, ROS and NETs appear to be highly context dependent. While excessive or persistent ROS production and dispersed NET formation can amplify tissue injury, expose autoantigens, and perpetuate chronic inflammation [[16], [17], [18], [19], [20]], short-lived and/or spatially confined ROS/NET formation can support the resolution of inflammation [15,21].

Experimental NOX2 agonists (i.e., chemical compounds activating NOX2) have been successfully employed to resolve chronic inflammation *in vivo* in murine models of inflammatory diseases [22]. However, they only act in conditions of fully functional NOX2 and therefore cannot alleviate chronic inflammation connected to NOX2-dysfunctionality. Furthermore, it is known that unspecific ROS amplifiers with anti-inflammatory effect, e.g., PEGylated d-amino acid oxidase [23], pioglitazone, and rosiglitazone [24] are able to induce NET formation in CGD-derived neutrophil granulocytes. However, the currently available ROS-amplifiers generate ROS unabashedly and systemically, which leads to high levels of oxidative stress and collateral damage of normal tissues, limiting their possibility for clinical application. Therefore, the search for alternative, safer approaches for the treatment of NOX2-deficiency-associated conditions is warranted [25].

N-Alkylaminoferrocene (NAAF)-based prodrugs (further pro-NAAFs) have been originally developed as anticancer agents. They are activated in the presence of the elevated ROS concentrations characteristic for the intracellular environment of cancer cells (1 to 10 μM) [26,27]. In contrast, in the intracellular environment of normal cells, the concentration of ROS is usually substantially lower (0.001-0.7 μM H_2_O_2_ [28]). Under the latter conditions, pro-NAAFs stay inactive. The mode of action of these prodrugs relies on the release of NAAF/NAAF^+^ drugs upon activation, which are catalysts of generation of highly reactive HO• from H_2_O_2_. Furthermore, p-quinone methide (QM) is released, which can alkylate nucleophilic biomolecules (e.g., GSH and thioredoxin reductase (TrxR)), thereby inhibiting the cellular antioxidant system [29,30]. Both NAAF/NAAF^+^ and QM act synergistically, thus leading to the multiplication of ROS.

Herein we provide proof-of-concept data obtained in cell-free settings, in experiments with primary murine (both wild type and NOX2-dysfunctional) and human (normal healthy donors and CGD patients) cells as well as *in vivo* results (a monosodium urate (MSU) crystal-induced model of chronic arthritis in NOX2-dysfunctional *Ncf1∗∗* mice), which confirm the feasibility of the application of pro-NAAFs for the correction of the inflammatory phenotype connected to insufficient activity of NOX2. Furthermore, our data suggest that the mode of action of pro-NAAFs includes inflammasome inhibition and the ROS-dependent induction of aggNETs.

## 2 Results

### 2.1 Pro-NAAFs multiply ROS in the presence of molecular oxygen

**Fig. 1:**
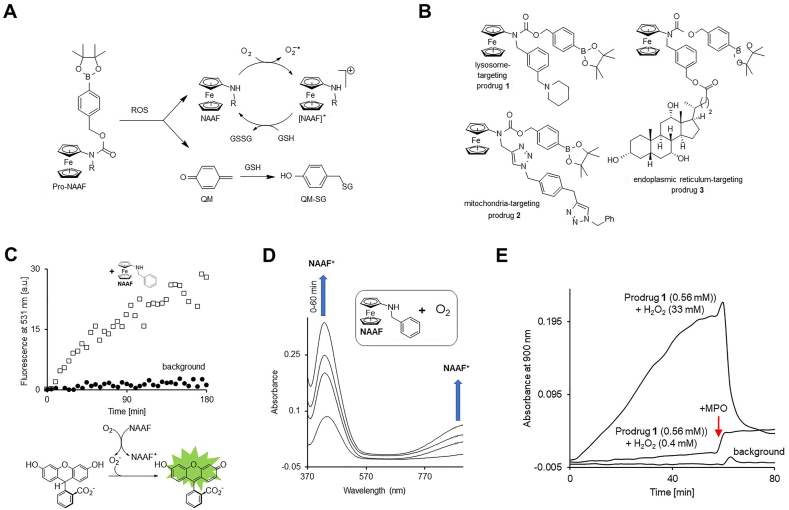
**Activation mechanism of N-alkylaminoferrocene (pro-NAAF) prodrugs and catalytic amplification of reactive oxygen species (ROS). (A)** The mechanism of activation of pro-NAAFs in the presence of ROS with formation of N-alkylaminoferrocene drugs (NAAFs) able to catalyze generation of ROS from oxygen (O_2_). GSH and GSSG: glutathione and glutathione disulfide, correspondingly; QM: para-quinone methide; QM-SG: QM adduct with glutathione. **(B**) Chemical structures of prodrugs **1**, **2** and **3** tested in this study. **(C)** NAAF catalyzes oxidation of dihydrofluorescein (O_2_•^-^, HO• probe) in the presence of oxygen. **(D)** NAAF is oxidized to NAAF^+^ in the presence of oxygen. Plots show the increase of the characteristic absorbance at 900 nm and increase of the UV-band of the ferrocene chromophore. **(E)** Kinetics of pro-NAAF activation in the presence of low and high amounts of ROS (H_2_O_2_). The reaction can be facilitated by addition of myeloperoxidase (MPO).

A structure of the parent pro-NAAF is shown in Fig. 1A. Here the ferrocene fragment is covalently attached to an electron-acceptor protecting group (arylmethyloxycarbonylamino). Therefore, the molecules of this type are relatively electron-deficient and therefore do not act as electron donors in the extracellular space or in cells [31,32]. Furthermore, the pro-NAAFs used in this study contain aryl boronic acid ester as a ROS-responsive triggering moiety (Fig. 1A and B). Its removal leads to formation of unstable phenol or aniline derivatives spontaneously undergoing 1,6-elimination with formation of substituted aminoferrocenes (NAAF), which are substantially more electron-rich than the parent pro-NAAF. Therefore, they act as electron donors for endogenous H_2_O_2_ and O_2_. This process results in the formation of highly reactive ROS species (HO• and O_2_•^-^), as confirmed for a representative aminoferrocene drug (Fig. 1C). During this reaction, ferrocenium cations (NAAF^+^) are formed and can be monitored by UV–visible spectroscopy through their characteristic absorbance at 900 nm (Fig. 1D). These cations can be reduced back by endogenous bulk reducing agents (GSH, ascorbate, and NADPH), thereby closing the catalytic cycle. Thus, the pro-NAAFs amplify already present ROS, with the kinetics of the reaction dependent on the concentration of pre-existing ROS. Under conditions of low ROS concentrations, the chemical reaction of prodrug **1** can be accelerated by the addition of myeloperoxidase (Fig. 1E).

### 2.2 Induction of ROS- and NET-formation by a series of selected Pro-NAAFs

To find the most efficient and safe way for ROS modulation in neutrophils leading to NET formation and inhibition of inflammation, we tested a series of diverse pro-NAAF-based ROS amplifiers. In particular, we selected representative prodrugs **1** [33], **2** [31], and **3** [34], which are known to affect functions of different intracellular organelles, specifically lysosomes (prodrug **1**), mitochondria (prodrug **2**) and endoplasmic reticulum (ER) (prodrug **3**) in cancer cells (Fig. 1B). The selected prodrugs increase intracellular ROS via different ways including lysosomal disruption and catalytic ROS production (prodrug **1**) [33], the depolarization of the mitochondrial membrane potential leading to the enhanced generation of both mitochondrial (mtROS) and total ROS (tROS, prodrugs **2** and **3**) [31,34], and the induction of ER stress resulting in the amplification of both mtROS and tROS (prodrug **3**) [34]. For prodrug **1** we developed an optimized synthesis protocol as an HCl salt (Suppl. Fig. S1), enabling the isolation of the compound with a purity exceeding 95%. This procedure is readily scalable, allowing the preparation of sufficient quantities of prodrug **1** for the extensive *in vivo* experiments reported in this study. Its fluorogenic version (Suppl. Fig. S4C) was obtained as described elsewhere [35]. Other prodrugs (**2**, **3**) were obtained using previously established protocols [31,34].

**Fig. 2:**
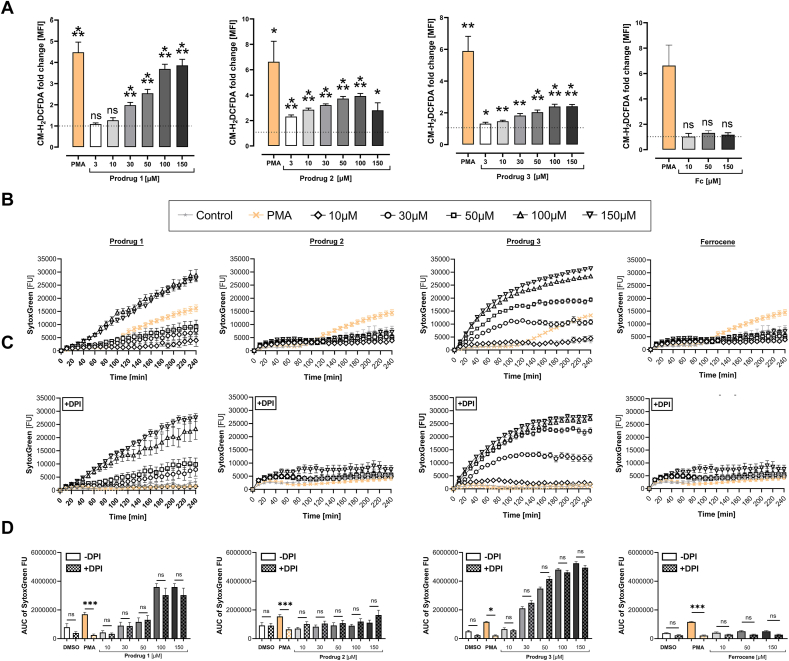
**(next page). Intracellular ROS accumulation and extrusion of extracellular DNA in human peripheral blood neutrophils treated with pro-NAAFs. (A)** Intracellular ROS levels after incubation of various concentrations of pro-NAAFs and ferrocene as a negative control compound (Fc). Bars show means and S.E.M. of fold changes over incubation with vehicle (1,5% DMSO). N = 6-8 individual normal healthy donors (NHDs). ∗p < 0.05, ∗∗p < 0.01, ∗∗∗p < 0.001, ns, not significant, One sample *t*-test. **(B**–**D)** Fluorescence curves (B, C) and area under the curves (D) of concentrations of extracellular DNA (shown by SytoxGreen fluorescence) in supernatants of human NHD neutrophils pretreated without (B) or with (C) diphenylene iodonium (DPI) to block NOX2 activity and incubated for 4 h with different concentrations of pro-NAAFs or Fc. ∗p < 0.05, ∗∗p < 0.01, ∗∗∗p < 0.001, ns, not significant; as determined by Friedman test with Dunn's multiple comparisons test. N = 12-15.

We first screened the three pro-NAAFs in neutrophils isolated from peripheral blood of healthy human blood donors (NHDs). All three NAAFs tested induced intracellular ROS accumulation in a dose-dependent way (Fig. 2A). Prodrug **1** had the strongest ROS-inducing effect. Prodrugs **2** and **3** also induced marked intracellular ROS accumulation, albeit at lower levels than prodrug **1**. Of note, the ROS-inducing capacity of prodrugs **2** and **3** peaked at 100 μM concentrations and were not further increased or were even decreased at 150 μM concentrations.

We then used release of extracellular DNA as a measure of induction of NETs by pro-NAAFs. After exposure to >100 μM of prodrug **1** and 30 μM prodrug **3** human PMNs released amounts of extracellular DNA similar to or above the DNA levels released after stimulation with the protein kinase C agonist phorbol-12-myristate-13-acetate (PMA), which triggers activation of NOX2 [36] (Fig. 2B). In contrast, prodrug **2** was comparably poor in induction of extracellular DNA release. The extracellular DNA release induced by pro-NAAFs was not affected by pretreatment of neutrophils with diphenylene iodonium (DPI) (Fig. 2C and D), demonstrating that NET formation occurred independently of NOX2. Other than pro-NAAFs the negative control ferrocene (Fc) did not induce intracellular ROS or extracellular DNA release.

Prodrug **3** induced a very sharp increase of extracellular DNA release, suggesting that other forms of cell death might be at work. Indeed, a flow cytometric analysis [37] at early timepoints revealed much higher numbers of stressed cells with reduced mitochondrial membrane potential, and also cells with destroyed membrane integrity upon incubation with Prodrug **3** than with the other two prodrugs (Fig. S2A).

**Fig. 3:**
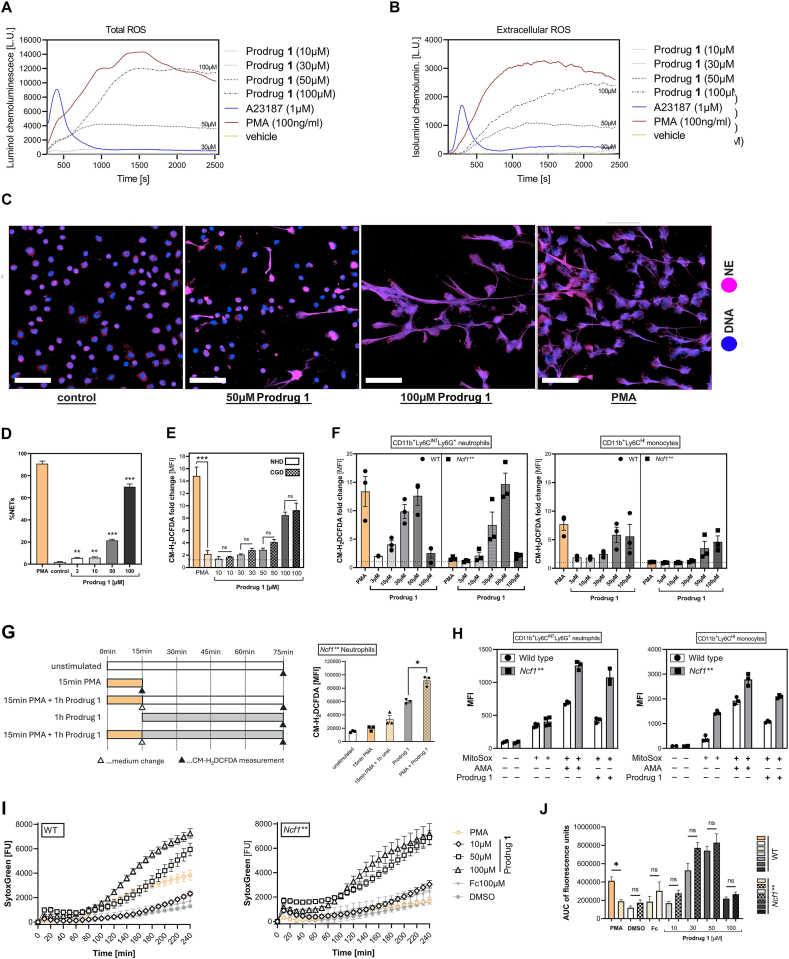
**ROS and NET formation induced by prodrug 1. (A, B)** Dynamics of total (A) and extracellular (B) ROS formation in human blood neutrophils, as determined by luminol and isoluminol luminescence, respectively. Curves show means from 3 normal healthy donors (NHDs). **(C, D)** Re-presentative images (C) and quantification (D) of NETs induced by prodrug **1** in human neutrophils. Scale bars, 100 μm ∗∗p < 0.01, ∗∗∗p < 0.001, as compared to DMSO control. ANOVA with Dunnet's multiple comparisons test. N = 5. **(E)** ROS production in neutrophils from human individuals with CGD and age- and sex-matched NHDs upon incubation with prodrug **1**. ∗∗∗p < 0.001, ns, not significant, as determined by ANOVA with Sidak's multiple comparisons test. N = 3 donors pr group. **(F)** Intracellular ROS accumulation in murine wild type (WT) and NOX2-dysfunctional (*Ncf1∗∗*) blood neutrophils and monocytes upon incubation with prodrug **1**. **(G)** Prodrug **1**-induced intracellular ROS production in *Ncf1∗∗-*derived blood neutrophils with or without preincubation with phorbol-12-myristate-13-acetate (PMA). N = 3 mice/group, ∗p < 0.05, paired *t*-test. **(H)** Accumulation of mitochondrial ROS measured by mitoSOX in murine WT and *Ncf1∗∗* neutrophils and monocytes upon incubation with prodrug **1** or antimycin A (AMA). **(I, J)** Trend curves (I) and area under the curves (AUC, J) of extracellular DNA release from prodrug **1**-treated murine WT and *Ncf1∗∗* blood neutrophils. ∗p < 0.05, ns, not significant, Kruskal- Wallis test with Dunn's multiple comparisons test. N = cells from 5 to 12 mice/group.

Due to its superior capacity to induce both strong ROS production and extracellular DNA release from human neutrophils, we selected prodrug **1** for further testing. Strong tROS production and extracellular ROS release by human neutrophils after incubation with prodrug **1**- concentrations from 50 μM was confirmed by luminol and isoluminol chemiluminescence, respectively (Fig. 3A and B). In these assays, ROS production induced by 100 μM prodrug **1** exhibited a slower dynamic but was more sustained than ROS induced by PMA. Co-incubation with the NOX2-inhibitor GSK2795039 [38] did not reduce tROS production measured by luminol (Fig. S3A), confirming the independence of prodrug **1**-induced ROS formation from NOX2. Prodrug **1** also induced ROS in human monocytes, and in CD3^+^ T cells (Fig. S3B). The induction of NETs in human blood neutrophils by prodrug **1** was confirmed by fluorescence microscopy (Fig. 3C and D, S3B). Furthermore, intracellular ROS accumulation and NET formation upon exposure to prodrug **1** were maintained in blood neutrophils from CGD patients (Fig. 3E–S3B).

Similar to human neutrophils, prodrug **1** also prompted intracellular ROS accumulation in neutrophils and other phagocytes from mouse blood (Fig. 3F). Comparably to humans, NOX2 was not needed for tROS or NET formation induced by prodrug **1**, as shown from results from neutrophils carrying a mutation in the regulatory subunit Neutrophil cytosolic factor 1 (*Ncf1)*, which renders Ncf1 unable to activate NOX2 [39,40].

In our experiments so far, prodrug **1** had induced ROS production from phagocytes capable of producing large amounts of ROS during a classical oxidative burst, but also from other cell types with supposedly lower inducible ROS-production capacity, such as T cells, raising the question of the selectivity of the compound towards cells with high ROS production. We therefore queried if prestimulation of cells to induce ROS production would result in a higher final prodrug **1**-induced ROS output than from naïve cells. To this end, we prestimulated *Ncf1∗∗-*derived neutrophils with PMA, with induces a strong oxidative burst downstream of protein kinase C-activation under conditions of normal NOX2 function [41], but which induced only mild cell activation and low ROS production without quickly triggering NETosis in NOX-dysfunctional *Ncf1∗∗* neutrophils (Fig. 3G). PMA-pre-stimulated *Ncf1∗∗* neutrophils exhibited higher levels of ROS accumulation after subsequent incubation with prodrug **1,** suggesting that the performance of prodrug **1** is quantitatively dependent on already present ROS.Incubation with the mitochondria-targeting fluorescent probe mitoSOX revealed considerable induction by prodrug **1** of mitochondrial ROS in blood-derived wildtype (WT) monocytes, but not WT neutrophils. Interestingly however, prodrug **1** did induce mitoROS in *Ncf1∗∗* neutrophils (Fig, 3H).

Finally, prodrug **1** also induced NET formation dose-dependently in blood neutrophils from both WT and *Ncf1∗∗* mice (Fig. 3I and J).

### 2.3 Prodrug 1-induced aggregated NETs degrade inflammatory mediators *in vitro* and *in vivo*

**Fig. 4:**
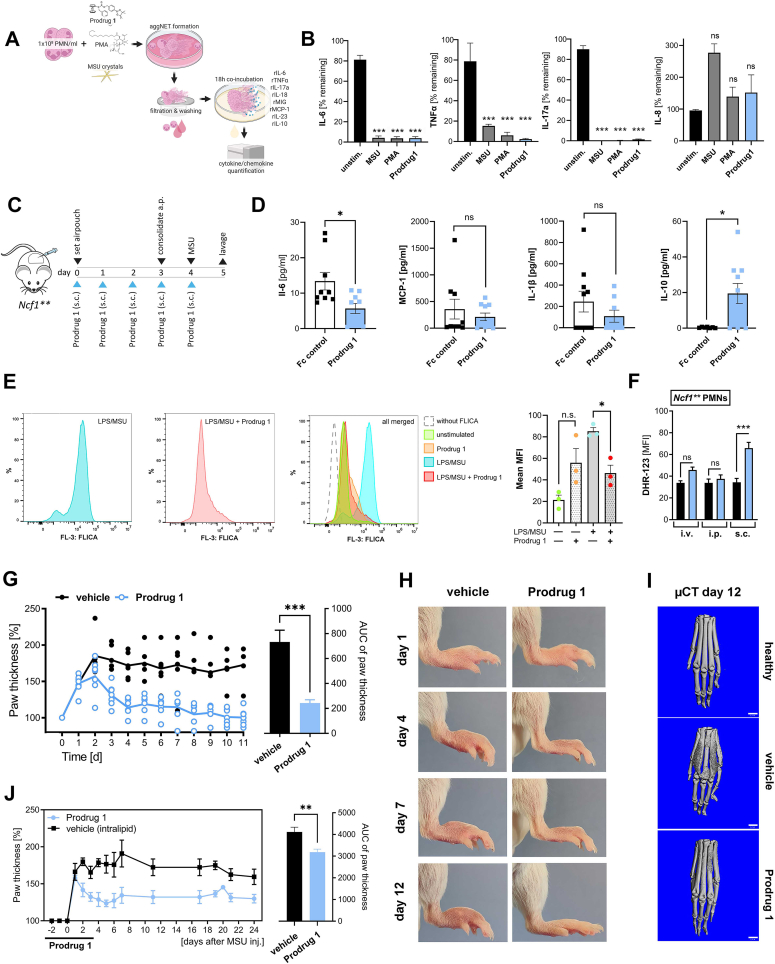
**(next page). Prodrug 1 triggers anti-inflammatory aggNET formation, reduces Monosodium urate (MSU) crystal-induced inflammasome activation, and induces resolution of MSU crystal-induced arthritis in mice**. **(A, B)** Degradation of inflammatory mediators by prodrug **1**-induced aggregated NETs. ∗∗∗p < 0001, as determined by ANOVA with Dunnett's multiple comparisons test. N = 2-6. **(C, D)** Treatment scheme (C) and (D) concentrations of inflammatory mediators in lavages from MSU crystal-injected air pouches after treatment of *Ncf1∗∗* mice with vehicle or prodrug **1**. ∗p < 0.05, Mann Whitney *U* test. **(E)** Flow cytometric quantification of caspase-1 enzymatic activity in human blood neutrophils by labeling with the fluorescent caspase-1 probe FAM-YVAD-FMK (FLICA) after stimulation with lipopolysaccharide (LPS) and MSU crystals and co-incubation with prodrug **1**. Shown are representative histogram plots (left) and results of quantification from 3 normal healthy blood donors (right). ∗p < 0.05, n.s., not significant, paired ANOVA with Sidak's multiple comparisons test. **(F)** ROS production in *Ncf1∗∗* neutrophils after intravenous (i.v.), intraperitoneal (i.p.) or subcutaneous (s.c.) treatment with vehicle (black bars) or prodrug **1** (blue bars). ∗∗∗p < 0.01, ANOVA with Sidak's multiple comparisons test. N = 8-10 mice. **(G**–**J)** MSU crystal-induced arthritis in *Ncf1∗∗* mice treated with prodrug **1**. **(G)** Arthritis curves and area under the curves (AUC) upon daily s.c. treatment with either vehicle or prodrug **1** from 2 days before arthritis induction until the end of the experiment. ∗∗∗p < 0.001, Student's *t*-test. N = 6-7 mice per group. **(H)** Representative photographs from mouse paws. **(I)** Representative micrographs from μCT. ∗∗∗p < 0.001, Student's *t*-test. **(J)** Arthritis curves and AUC upon cessation of prodrug **1** treatment 3 days after arthritis induction. ∗∗p < 0.01, Student's *t*-test. N = 5 mice per group.

To investigate if NETs induced by prodrug **1** have anti-inflammatory properties, we formed aggregated NETs (aggNETs) by incubating human neutrophils from peripheral blood with either prodrug **1**, PMA or monosodium urate (MSU) crystals. AggNETs induced by prodrug **1** exhibited degradative properties on both externally added recombinant cytokines and chemokines (Fig. 4A and B, Fig S4A), similar to aggNETs induced by PMA, MSU crystals, and other known NET inducers [13]. Similarly to previous results, only IL-8 was not reduced in concentration in the supernatants after incubation with aggNETs, since it is released in large amounts by aggNETs [12]. In *Ncf1∗∗* mice, pretreatment with prodrug **1** reduced the concentrations of pro-inflammatory mediators in an airpouch model as compared to treatment with Fc control (Fig. 4C and D).

### 2.4 Prodrug 1 treatment dampens MSU crystal-induced inflammasome activation in neutrophils and induces resolution of chronic arthritis in NOX2-dysfunctional mice

MSU crystal induced arthritis is driven by inflammasome activation [42,43]. To assess if treatment with prodrug **1** affects inflammasome activation, we pretreated human blood neutrophils successively with LPS and MSU crystals *in vitro*, followed by a treatment with prodrug **1**. We then assessed caspase 1 activity by FLICA. We observed that co-incubation with prodrug **1** significantly inhibited caspase 1 activity induced by LPS/MSU crystals (Fig. 4E). Of note, incubation with prodrug **1** alone showed a trend to induce caspase 1-activation. In line with the results from caspase 1-activation, IL-1β concentrations were elevated in supernatants from neutrophils incubated with prodrug **1** alone, while co-incubation with prodrug **1** reduced IL-1β release in LPS/MSU crystal-stimulated neutrophils (Fig. S4B),

Finally, to assess if *in vivo* application of prodrug **1** could rectify dysfunctional ROS production and affect arthritis we tested intracellular ROS accumulation in mouse neutrophils after intravenous (i.v.), intraperitoneal (i.p) or subcutaneous (s.c.) pretreatment with prodrug **1**. S.c., but not i.v. or i.p. injections significantly enhanced intracellular levels of ROS in *Ncf1∗∗* neutrophils (Fig. 4F). Intriguingly, daily s.c. injection with prodrug **1** induced resolution of MSU crystal-induced arthritis in *Ncf1∗∗* mice (Fig. 4G and H), which usually takes a chronic course in this strain [12,14]. Resolution of arthritis by prodrug **1** was accompanied by protection from leukocyte infiltration into the joint and arthritis-related bone destruction (Fig. 4I–S4C, S4D) and was also maintained when prodrug **1**-injections were discontinued 3 days after induction of arthritis with MSU crystals (Fig. 4J).

To test the whereabouts of prodrug **1** after s.c. injection in arthritis, we used a curcumin-based colorimetric assay [44], which detects the boronic acid ester in unconjugated prodrug **1**. Higher boron concentrations were found in extracts from prodrug **1**-injected than from non-injected skin areas (Fig. S4E). Interestingly, lower boron concentrations were measured in the arthritic paw injected with MSU crystals than in the non-inflamed contralateral paw, suggesting enhanced consumption of prodrug **1** in inflamed tissue. These results were confirmed by Infrared spectroscopic *in vivo* imaging using a fluorogenic version of prodrug **1** (Fig, S4F, S4G), where prodrug **1**-BODIPY fluorescence decrease was observed in the MSU crystal-injected paw compared to the contralateral paw injected with only saline. This decrease was associated with compound consumption (Fig. S4F).

### 2.5 Transcriptomic analysis of effect of prodrug 1 *in vivo* treatment

**Fig. 5:**
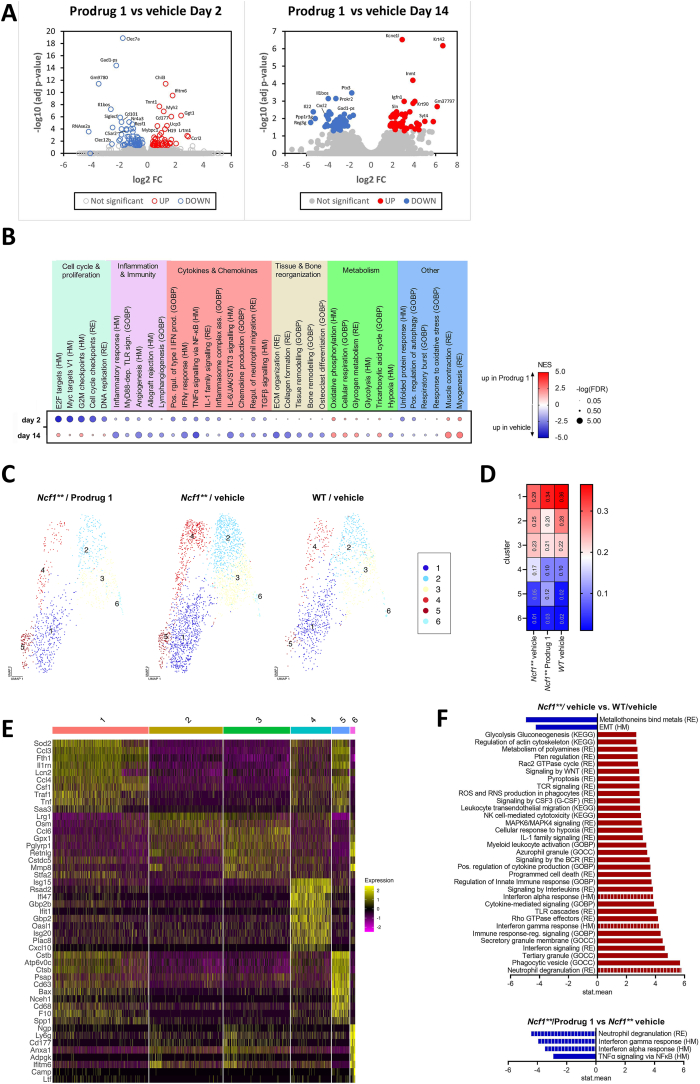
**Transcriptomic analysis from paws of mice treated with prodrug 1**. **(A**–**B)** Bulk RNA-Seq in whole paws of *Ncf1∗∗* mice 2 or 14 days after initiation of monosodium urate (MSU) crystal-induced arthritis and treated with daily s.c. injections of either prodrug **1** or vehicle (intralipid). **(A)** Volcano plots of differential gene expression in paws of prodrug **1**- vs vehicle-treated mice on day 2 and day 14, respectively (d2/prodrug **1**: n = 5 mice, d2/vehicle: n = 3 mice, d14/prodrug **1**: n = 4 mice, d14/vehicle: n = 4 mice). **(B)** Gene set enrichment analysis (GSEA) in paws showing pathways affected by treatment with prodrug **1** two and 14 days after initiation of MSU crystal-induced arthritis, respectively. FDR, false discovery rate; NES, Normalized enrichment score. **(C–F)** Single cell RNA-Seq from paws of prodrug **1**- and vehicle-treated *Ncf1∗∗* and wildtype (WT) mice 5 days after injection of MSU crystals. **(C)** Non-linear dimensional reduction Uniform Manifold Approximation and Projection (UMAP) plots of neutrophil clusters split by different conditions. **(D)** Relative abundances of cell clusters between different conditions. **(E)** Heatmap displaying the top 10 differentially expressed genes (DEGs) for each cluster identified at a resolution of 0.5. Expression levels are shown as scaled normalized counts, with rows representing genes and columns representing individual cells grouped by cluster. The color scale represents the z-score of gene expression, ranging from low (purple) to high (yellow) expression. **(F)** Gene set enrichment analysis of neutrophil clusters was performed using the Generally Applicable Gene-set Enrichment (GAGE) R package. Shown, are selected differentially regulated pathways (q value < 0.1) in neutrophils from vehicle-treated *Ncf1∗∗* vs vehicle-treated WT mice (upper panel) and from prodrug **1**-treated vs vehicle-treated *Ncf1∗∗* mice (lower panel). Pathways differentially regulated in both comparisons are shown with striped bars. GOBP, gene ontology biological pathways; GOCC, gene ontology cellular components; KEGG, Kyoto Encyclopedia of Genes and Genomes; RE, reactome. The x-axis represents the stat.mean, indicating the overall direction and magnitude of the pathway change (effect size) based on gene-level log2 fold-changes. Positive values (red) denote up-regulated pathways, while negative values (blue) denote down-regulated pathways.

To shed some light on mechanistic aspects behind the induction of inflammatory resolution by prodrug **1**, we performed transcriptomic analysis of paws from prodrug **1** or vehicle-treated mice. Gene set enrichment analysis (GSEA) of bulk RNA-Seq (Fig. 5A and B) revealed strong downregulation of pathways connected to cell proliferation and cytokine/chemokine response in prodrug **1**-treated mice during early MSU crystal-induced arthritis, despite negligible differences in paw swelling between the treatment and the control group. These results suggest that treatment with prodrug **1** affects arthritis already in an early, acute stage, initializing later resolution of inflammation. While pathways related to cellular proliferation, the inflammatory and immune response were down at day 2, metabolic pathways such as oxidative phosphorylation were upregulated upon treatment with prodrug **1**. In line with that, upstream regulator analysis by Ingenuity pathway analysis confirmed the immune-modulatory and attenuating effect of prodrug **1** in early inflammation (Supplementary Table 1). Hence, the negative regulator CISH was activated, suggesting prodrug **1**-induced inhibition of JAK-STAT cytokine signaling. In contrast, CD44, a key mediator of leukocyte adhesion and migration, was reduced upon treatment, consistent with decreased immune cell infiltration into inflamed tissue.

During chronic arthritis, at day 14 after injection of MSU crystals, GSEA revealed sustained suppression of the cytokine/chemokine and total inflammatory response together with ongoing metabolic activation in the paws of mice treated with prodrug **1**. Suppression of pathways connected to tissue and bone remodeling confirmed that treatment with prodrug **1** confers protection from arthritis-related bone destruction and functional decline.

Accordingly, upstream regulator analysis at day 14 predicted strong inhibition of innate immune signaling pathways and promotion of resolving pathways by prodrug **1**. Key inflammatory regulators including MYD88, a central adaptor for Toll-like receptor and IL-1 signaling, and the chemokines CCL2 and CCL5 were predicted to be inhibited upon treatment, indicating suppression of myeloid cell activation and recruitment. In contrast, the negative cytokine regulator CISH and the anti-inflammatory cytokine IL10 were activated (Supplementary Table 2).

To transcriptomically dissect the impact of prodrug **1**-treatment on cytosolic and mitochondrial ROS production, we re-assessed expression changes of cytosolic and mitochondrial ROS regulators [45] in our bulkRNA-Seq datasets from paws of prodrug **1**- and vehicle-treated *Ncf1∗∗* mice (Fig. S5A and B, Supplementary Table 3). These analyses showed that on day 2 after arthritis induction pro-cytosolic ROS regulators exhibited a significant shift towards a high expression in paws from prodrug **1**-treated mice (p = 0.01614, two-sided *t*-test), whereas anti-cytosolic ROS regulators were not significantly shifted in either direction. On day 14, neither pro- nor anti-cytosolic ROS regulators showed significant shifts (Fig. S5A). With regards to mitochondrial ROS, expression of both pro- and anti-mitochondrial ROS regulators was robustly upregulated in paws from prodrug **1**- treated as compared to paws from vehicle-treated *Ncf1∗∗* mice at both day 2 (p = 0.00124 for pro-mitochondrial and p = 0.0379 for anti-mitochondrial ROS regulators, respectively) and day 14 (p = 0.00116 and 0.01324, respectively) (Fig. S5B).We next used single-cell (sc) RNA-seq of whole paws during acute MSU crystals-induced arthritis enriched for neutrophils (Fig. 5C–F, S5C-D) to link our findings to subpopulations of cells. Single cell transcriptomes were restricted to neutrophils and clustered. Consistent with previous results, we identified a continuous distribution of neutrophils, suggesting ongoing molecular evolution in the inflamed joint [46] (Fig. 5C). We divided neutrophils into six populations for quantification and identification of marker genes. Splice kinetics analysis with scVelo confirmed a temporal progression of cluster 6 through 3, 2, 4, 1 and finally 5. Cluster 1 was the most prominent neutrophil phenotype in WT paws (Fig. 5D). By contrast, in *Ncf1∗∗* mice, cluster 4 was expanded and cluster 1 diminished, highlighting a shift towards a less mature neutrophil pool in the joint. Treatment of *Ncf1∗∗* mice with prodrug **1** restored the balance of neutrophil phenotypes observed in WT mice. Concordantly, GSEA in neutrophils uncovered that the most strongly downregulated pathways upon treatment with prodrug **1** were connected to neutrophil degranulation, TNFα, and IFN signaling (Fig. 5F), the latter of which had also been associated with neutrophil maturation [46] and the neutrophil phenotype in synovial fluid of arthritic individuals [47]. Comparing the prodrug **1**-induced changes in *Ncf1∗∗* neutrophils with the differences between *Ncf1∗∗* and WT neutrophils, prodrug **1**-treatment shifted the *Ncf1∗∗* neutrophil phenotype towards the transcriptome of WT neutrophils.

### 2.6 Prodrug 1-treatment shifts protein oxidation towards an inflammation-resolving profile

**Fig. 6:**
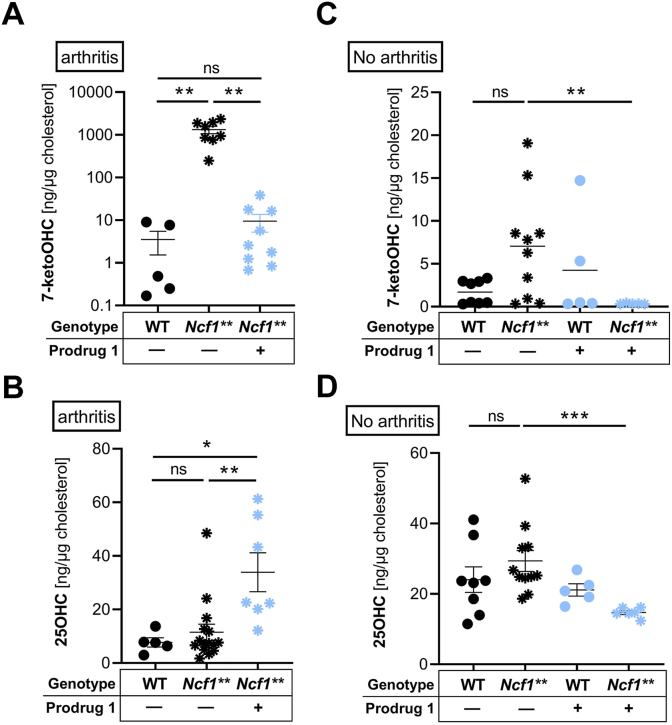
**Concentrations of oxysterols in mouse plasma. (A)** Plasma concentrations of 7-ketoOHC in wild type (WT) and *Ncf1∗∗* mice at day 14 after induction of MSU crystal-induced arthritis and with/without daily subcutaneous injections of prodrug **1**. **(B)** Plasma concentrations of 25OHC in WT and *Ncf1∗∗* mice at day 14 after induction of MSU crystal-induced arthritis and with/without daily subcutaneous injections of prodrug **1. (C)** Plasma concentrations of 7-ketoOHC in WT and *Ncf1∗∗* mice after 14 days treatment with prodrug **1**, without induction of arthritis, and in untreated healthy control mice. **(D)** Plasma concentrations of 25OHC in WT and *Ncf1∗∗* mice after 14 days treatment with prodrug **1**, without induction of arthritis, and in untreated healthy control mice. ∗p < 0.05, ∗∗p < 0.01, ∗∗∗P < 0.001, as determined by Kruskal Wallis test with Dunn's post hoc test.

The anti-inflammatory action of prodrug **1** by induction of aggNET formation has to be balanced against the eventual induction of oxidative stress. We observed an enhanced abundance of carbonylated proteins in sera of *Ncf1∗∗* mice treated with prodrug **1** (Fig. S6A). To assess markers of protein oxidation in more detail we measured concentrations of various oxysterols in murine plasma. 7-ketoOHC, which is connected to inflammation and many diseases [48,49] was strongly reduced in *Ncf1∗∗* mice by treatment with prodrug **1** from the enhanced levels observed during chronic arthritis, while the anti-inflammatory 25OHC was raised (Fig, 6A, 6C). Since these changes could also be caused by the reduced clinical disease upon Prodrug 1-treatment we also tested oxysterol levels upon treatment in healthy, non-arthritic mice. Here, Prodrug 1-treatment reduced both 7-ketoOHC and 25OHC levels in plasma of *Ncf1∗∗* mice while the levels were not affected in WT animals (Fig. 6B and D).

## 3 Discussion

In this study, we demonstrate that N-alkylaminoferrocene–based prodrugs (pro-NAAFs) function as NOX2-independent amplifiers of reactive oxygen species (ROS) capable of restoring inflammation-resolving neutrophil functions under conditions of NOX2 deficiency. Prodrug **1** triggered robust neutrophil extracellular trap (NET) formation in both human and murine neutrophils independently of NOX2 activity, including neutrophils derived from patients with chronic granulomatous disease (CGD) and from NOX2-dysfunctional *Ncf1∗∗* mice. The induced NETs aggregated into anti-inflammatory aggNETs with potent cytokine- and chemokine-degrading capacity. *In vivo*, subcutaneous administration of prodrug **1** lowered inflammatory mediator levels in a murine airpouch model, promoted resolution of monosodium urate (MSU) crystal-induced chronic arthritis in *Ncf1∗∗* mice, prevented bone destruction, and normalized neutrophil transcriptional programs towards a wild-type–like profile. Collectively, these findings identify pro-NAAFs as a pharmacological strategy to restore defective ROS-dependent inflammatory resolution in NOX2-deficient settings.

CGD is a primary immunodeficiency, estimated to affect about 1 in 200,000–250,000 people worldwide, with substantial clinical impact caused by mutations affecting components of the NOX2 complex, resulting in defective phagocyte-derived ROS production [4,50,51]. While CGD is classically associated with recurrent and severe bacterial and fungal infections, impaired NOX2 activity also predisposes patients and animal models to chronic sterile inflammation and autoimmunity. Accordingly, individuals with deficient NOX2 frequently develop granulomatous lesions, inflammatory bowel disease–like colitis, arthritis, Guillain-Barré syndrome, and lupus-like manifestations [6,7,10,[52], [53], [54], [55]]. Similar phenotypes are observed in rodents with partial or complete NOX2 dysfunction, including *Ncf1*-mutant mice, which display exaggerated and non-resolving inflammatory responses in models of arthritis and other autoimmune and inflammatory conditions [5,8,9,12,56]. Mechanistically, insufficient ROS production compromises NET formation, phagocytic clearance, local cytokine degradation by aggNETs, and immune regulatory feedback loops that normally promote the resolution of inflammation [[11], [12], [13],^57^].

Current management of CGD-associated inflammation relies largely on broad anti-inflammatory and immunosuppressive strategies, including corticosteroids, biologics targeting TNF-α or IL-1, and, in severe cases, hematopoietic stem cell transplantation [[58], [59], [60]]. Experimental approaches to compensate for defective ROS generation, such as NOX2 agonists or enzymatic ROS producers (e.g., PEGylated d-amino acid oxidase), generally require residual NOX2 activity or induce systemic oxidative stress, limiting their applicability and safety [25].

Our findings suggest that pro-NAAFs can overcome these limitations by acting as activation-dependent, NOX2-independent ROS amplifiers. Prodrug **1** preferentially amplifies ROS production in activated inflammatory environments, induces NET and aggNET formation, inhibits crystal-induced inflammasome activation, and restores inflammation-resolving pathways without requiring functional NOX2. Its *in vivo* efficacy in a chronic arthritis model and the normalization of neutrophil transcriptional trajectories support the translational potential of this approach for CGD-associated and other NOX2-linked inflammatory diseases.

Prodrug **1** had previously been shown to mostly target lysosomes as the major ROS-rich organelles in cancer cells [61], while we show here a considerable induction of ROS production by prodrug **1** in mitochondria. These findings demonstrate that (especially NOX2-dysfunctional) mitochondria of phagocytes can be considered ROS-rich environments, in which prodrug **1** is more readily activated.

Furthermore, our results show that prodrug **1** reduces MSU crystal-induced inflammasome activation directly in neutrophils. This is particularly interesting because mitochondrial ROS are often seen as permissive or amplifying signals for NLRP3 activation. The new data therefore argue against a model in which prodrug **1**-induced mtROS automatically enhances inflammasome activity. Rather, prodrug **1** may create a redox state that is, in sum, functionally suppressive for inflammasome execution, for example by impairing cellular metabolism, phagocytosis/crystal handling, lysosomal damage responses, inflammasome assembly, caspase-1 enzymatic activity, or general cell integrity.

At the same time, the trend toward increased inflammasome activity with prodrug **1** alone suggests that prodrug **1** may provide a weak danger-like signal by itself, consistent with its ability to induce mitochondrial oxidative stress. However, this effect seems comparably modest, whereas the dominant effect in the LPS/MSU setting is inhibitory.

Also the selectivity of prodrug **1** should be interpreted as relative rather than absolute. The observed activation of prodrug **1** in naïve neutrophils, monocytes, and even T cells *in vitro* is compatible with the presence of basal redox activity in these cell types. However, the higher ROS output after PMA prestimulation and the increased mitoSOX signal in NOX2-dysfunctional cells indicate that prodrug **1** responsiveness scales with the overall redox environment and available oxidative inputs, rather than depending exclusively on canonical NOX2-derived oxidative burst activity. This model also explains why CGD-derived neutrophils remain responsive: despite impaired NOX2 activity, their total basal redox state is usually not lower and may be supported by increased mtROS [62].

A central concern for ROS-based therapies is the risk of harmful oxidative stress. Pro-NAAFs differ fundamentally from conventional ROS-inducing agents by amplifying pre-existing ROS rather than generating ROS indiscriminately. Our data show that although prodrug **1** treatment increased protein carbonylation *in vivo*, this was accompanied by a shift towards an inflammation-resolving oxysterol profile, characterized by reduced levels of the pro-inflammatory derivative 7-ketocholesterol and increased or preserved concentrations of anti-inflammatory 25-hydroxycholesterol. These toxicity concerns should also be considered in the broader context of the dual roles of ROS and NETs, which can also act as drivers of autoimmunity, tissue damage and inflammation if their production is not spatially and temporally tightly controlled. Thus, the therapeutic aim of pro-NAAFs is not indiscriminate ROS enhancement, but a tailored amplification of pre-existing redox activity to restore local resolving functions, particularly in NOX2-dysfunctional inflammatory settings. In contrast to systemic ROS amplifiers such as thiazolidinediones or enzymatic oxidases, pro-NAAFs are preferentially activated within inflamed tissues, as evidenced by their enhanced consumption in arthritic as compared to non-inflamed contralateral paws. This spatial and functional restriction likely limits collateral tissue damage and distinguishes pro-NAAFs from ROS-inducing agents currently available on the market.

On a molecular level, pro-NAAFs could potentially be applied for treatment of pathologic conditions characterized by NOX2 deficiency in neutrophils (e.g., in autoimmune and chronic inflammatory conditions) with comparably low side effects due to the following considerations: it has been documented that the concentration of mitochondria-derived ROS (mtROS: O_2_^•-^ is initially formed from O_2_ and then dismutated to more stable H_2_O_2_) in resting CGD-derived neutrophils is only moderately elevated compared to normal neutrophils [62], in contrast to the strong increase of mtROS in cancer cells (>10 fold [63]). Therefore, it would be expected that pro-NAAFs are not efficiently activated in resting CGD-neutrophils. Upon activation after pathogen challenge or exposure to sterile inflammatory triggers, ROS levels in these neutrophils remain relatively moderate due to the absence of functional NOX2. However, myeloperoxidase (MPO) is released from primed CGD-neutrophils to a similar extent as in normal neutrophils [64] and other NOX and DUOX enzyme complexes are active. In the presence of MPO, mtROS (H_2_O_2_) is converted to the more potent oxidants HOCl and HO•, both of which are expected to activate the pro-NAAFs efficiently with formation of the active NAAF drug [65]. Accordingly, our data show increased pro-NAAF catalyzed ROS generation in the presence of MPO. In cancer cells this drug converts abundantly present H_2_O_2_ to HO•, thereby increasing oxidative stress [25,61]. In contrast, in CGD-derived neutrophils H_2_O_2_ is not available in large quantities [62]. Therefore, the latter mechanism of ROS amplification may be less dominant. However, the electron-rich NAAF drug can potentially donate an electron to molecular oxygen (O_2_) with the formation of a superoxide anion radical (O_2_^•-^), which subsequently undergoes spontaneous or superoxide dismutase–catalyzed dismutation to O_2_ and H_2_O_2_. In this way, a local pool of H_2_O_2_ may be generated, thereby enabling the NAAF-induced amplification of ROS analogous to that described for cancer cells. Hence, the NAAF-prodrug would act as a NOX2-mimetic selectively engaged in activated CGD-derived neutrophils, restoring functional responses such as NET formation and thereby contributing to resolution of inflammation.

However, several limitations and open questions of our study should be acknowledged. First, while the murine MSU-induced arthritis model recapitulates key aspects of chronic inflammation associated with NOX2 deficiency, it does not capture the infectious susceptibility characteristic of CGD. Second, long-term safety, dosing regimens, and potential cumulative oxidative damage require further investigation, and although prodrug **1** showed favorable tissue selectivity, comprehensive pharmacokinetic and toxicological studies are necessary prior to clinical translation. The route-dependent activity of prodrug 1 is such a pharmacokinetic and pharmacodynamic open question. The absence of ROS-inducing activity after intraperitoneal or intravenous injection likely reflects rapid dilution, clearance, and/or oxidative deactivation once the compound enters systemic circulation [66]. In contrast, subcutaneous administration may create a local depot that prolongs exposure and contact with target cells before systemic deactivation. The current formulation should therefore be viewed as most suitable for local or subcutaneous delivery, whereas clinical translation will require dedicated plasma-stability, tissue-distribution, dose-escalation, and formulation studies.

Of note, relatively high micromolar concentrations of prodrug 1 were required *in vitro* to induce ROS levels comparable to PMA. However, PMA is a somewhat artificial very strong NOX2-dependent positive control and therefore does not represent a clinically meaningful potency benchmark. More importantly, the prodrug **1** concentrations used in our study were sufficient to produce measurable biological effects after subcutaneous administration *in vivo*. While future medicinal-chemistry optimization will be required to increase potency and improve formulation properties, the present data show that biologically active local concentrations can be achieved without obvious acute toxicity.

Finally, although our study was focused on neutrophils and our *in vitro* data on cytokine degradation and inflammasome inhibition, together with previous work showing anti-inflammatory effects of neutrophils in crystal-induced inflammation, support an important contribution of neutrophil-derived aggNETs [12,14,67], the *in vivo* action of prodrug **1** cannot be assigned exclusively to neutrophils or NETs on the basis of the current experiments. Prodrug **1** was shown to also affects monocytes and other leukocyte populations *in vitro*, and effects on classical or non-classical innate immune cells likely contribute to the reduced arthritis phenotype.

## 4 Methods

### 4.1 Chemistry/formulation

#### 4.1.1 Improved synthesis of prodrug 1

Synthesis of the selected prodrug **1** was reported in 2017 [61]. The overall yield is below 10%, which results in difficult and time-consuming purifications as well as problems with scaling up. Moreover, prodrug **1** is obtained as oil, which is not convenient to handle and has a limited stability at 22°C. Since we required larger quantities of prodrug **1** for the *in vitro* and especially *in vivo* experiments, we decided to optimize the synthetic protocol to solve the mentioned above problems (further details in the Supplementary information). In the previously reported synthesis, first, the key intermediate S2 is obtained by alkylation of piperidine with 1,3-bis(chloromethyl)benzene (S1) followed by the chromatographic purification of the almost statistical mixture consisting of the starting material S1, the desired mono-substituted product (S2) and the bis-substituted side product. Due to the challenging purification, the yield of this reaction is only 23%. We explored the alternative pathway outlined in Supplementary Fig. S1. This new approach includes four rather than one synthetic steps. However, all of them do not require chromatographic purification and the whole sequence of the reactions can be conducted within less than 3 days. The key improvement here is in conducting the challenging desymmetrization reaction in the first step (*b*, Fig. S1A), which delivers the desired product with an excellent yield of 85%. The overall yield of the intermediate S2 was 75% that is a substantial improvement over the previously reported procedure.

In the next step (Fig. S1B) intermediate S6 is alkylated by S2. In the original procedure tetrabutylammonium iodide (TBAI) is used to activate S2 *in situ* to the corresponding iodo-derivative. The conversion of S6 is practically quantitative in this reaction. However, the separation of TBAI and the final product by chromatography is difficult. Moreover, the side product S7, formed in small quantities, has similar polarity to prodrug **1**, which further hampers chromatographic purification and leads to the loss of substantial amounts of the product. We observed that improvement can be achieved by the replacement of TBAI with lipophilic inorganic iodide CsI. The latter reagent can be easily separated from the product by aqueous extraction. Furthermore, the formation of the side product S7 could be minimized by optimization of the reaction time. Under the optimal conditions the yield of prodrug **1** in the final step reaches 80%, whereas the overall yield of the prodrug **1** starting from the intermediate S1 is 60%. The product is isolated as HCl salt. It is yellow and stable at 22°C as a powder (Fig. S1C), in contrast to the oily and less stable prodrug obtained by using the original protocol. We applied this developed procedure to obtain 3.56 g of the product with >95% purity.

In contrast to the previously used free base [61], the new product is isolated as a salt and can be solubilized in Intralipid after ultrasonication (Fig. S1C). We confirmed that both compounds exhibit the same (p = 0.8097, Student's *t*-test, Fig. S1) cell toxic effect towards the human cancer cell line A2780: prodrug **1**: IC_50_ = 6.3 ± 1.8 μM, N = 7; prodrug **1**∗HCl: IC_50_ = 5.9 ± 2.9 μM, N = 2.

#### 4.1.2 Synthesis

Intermediate **S4.** Intermediate **S4** was prepared as previously described [61] with few modifications as described below. HCl (18.5 ml, 12 M) was added at 22°C to a stirred suspension of 1,3-benzenedimethanol **S3** (5 g, 36.2 mmol) in toluene (180 ml). The resulting solution was stirred overnight at 22°C. Then, the solution was washed with water and aqueous NaHCO_3_, the organic phase was separated, dried over MgSO_4_ and the solvent was evaporated to afford **S4** as colorless oil (5 g, 85% yield). ^1^H NMR (300 MHz, CDCl_3_) *δ* (ppm) 7.41 – 7.30 (m, 4H), 4.71 (s, 2H), 4.60 (s, 2H), 1.68 (s, 1H).

Intermediate **S5**. **S4** (3.00 g, 19.1 mmol, 1 eq) was dissolved in acetonitrile (30 ml). To the solution K_2_CO_3_ (5.30 g, 38.3 mmol, 2 eq) and piperidine (1.71 g, 20.1 mmol, 1.05 eq) were added. The solution was stirred overnight, and the solvent was removed using rotary evaporator. The residue was partitioned between water and EtOAc. The organic phase was collected, dried over MgSO_4,_ and evaporated to give **S5** as viscous oil. Yield: 3.53 g (90%). ^1^H NMR (300 MHz, CDCl_3_) *δ* (ppm) 7.35 – 7.24 (m, 4H), 4.70-4.68 (d, J = 4.59 Hz, 2H), 3.51 (s, 2H), 2.41 (t, J = 4.15 Hz, 4H), 1.78 (t, J = 5.85 Hz, 1H), 1.60 (p, J = 5.54 Hz, 4H), 1.45 (q, J = 4.87 Hz, 2H).

Intermediate **S2. S5** (3.53 g, 17.2 mmol, 1 eq) was dissolved in HCl (45 ml, 12 M) and stirred overnight. The solution was evaporated and dried *in vacuo* (0.01 mbar) to give **4** as white crystals. Yield: 4.48 g (98%). ^1^H NMR (300 MHz, CDCl_3_) *δ* (ppm) 12.31 (s, 1H), 7.62 – 7.36 (m, 4H), 4.55 (s, 2H), 4.05 (d, J = 4.36 Hz, 2H), 3.37 (d, J = 11.86 Hz, 2H), 2.51 (q, J = 8.52 Hz, 2H), 2.24 (q, J = 12.89 Hz, 2H), 1.79 (t, J = 18.44 Hz, 2H), 1.29 (q, J = 11.50 Hz, 2H).

HCl salt of prodrug **1**. 4-(N-Ferrocenylaminocarbonyloxymethyl)phenylboronic acid pinacol ester **S6** (3.00 g, 6.5 mmol, 1 eq) was dissolved in anhydrous N,N-dimethylformamide (DMF, 43.37 ml, 0.15 M) and mixture of solid Cs_2_CO_3_ (5.36 g, 20 mmol, 3 eq) and CsI (1.69 g, 6.5 mmol, 1 eq) was added in one portion at 22°C. After 15 min **S2** (1.78 g, 6.8 mmol, 1.05 eq) was added in one portion at 22°C. The mixture was left stirring overnight. When the reaction was complete (monitored by LCMS) all volatiles were removed *in vacuo* (0.01 mbar) and CH_2_Cl_2_ (DCM) was added to the residue. The obtained suspension was filtered, and the DCM was removed using rotary evaporator. The remaining crude material was subjected to column chromatography (SiO_2_, DCM/MeOH, TEA = 10/1, 1%, Rf 0.47). The fractions containing the product were evaporated and dried. The product was re-dissolved in DCM and acidified with the solution of dioxane/HCl (4 M) until pH was 1. The solvents were removed using rotary evaporator and the remaining slurry was suspended in isopropanol. The suspension was stirred overnight and filtered. The solid obtained was dried *in vacuo* (0.01 mbar) overnight to give prodrug **1**∗HCl as yellowish powder (Fig. S1). Yield: 3.56 g, (80%). ^1^H NMR (400 MHz, DMSO-d6, Fig. S2) *δ* (ppm) 11.00 (s, 1H), 7.65 – 7.33 (m, 8H), 5.21 (s, 2H), 4.96 (s, 2H), 4.43 (s, 2H), 4.20 (s, 2H), 4.11 (s, 5H), 4.01 (s, 2H), 3.18 (s, 2H), 2.74 (s, 2H), 1.79 – 1.70 (m, 6H), 1.29 (s, 12H). ^13^C NMR (100 MHz, DMSO-d6, Fig. S3): *δ* (ppm) 153.80, 139.44, 138.78, 134.16, 129.67, 129.00, 128.58, 127.68, 126.84, 126.59, 100.55, 83.37, 68.51, 66.48, 63.92, 62.03, 58.46, 52.60, 51.13, 24.37, 21.78, 21.19.

#### 4.1.3 Synthesis and properties of prodrug 3 (fluorogenic version of prodrug 1) and control 4

Fluorogenic prodrugs allow investigating their mechanism of action directly in live cells and *in vivo* by using flow cytometry and fluorescence microscopy. However, it is not trivial to convert prodrugs to their fluorogenic versions, since the fluorescent moieties often affect the solubility in aqueous solution, intracellular distribution, and other important prodrug properties. For example, though the fluorogenic prodrug **1** has been already reported,^18^ its properties are not ideally matching the parent prodrug **1**. This includes its very low solubility in aqueous buffered at pH 7 solutions and low stability in DMSO stock solutions that prevented their applications in *in vivo* studies. Therefore, we prepared alternative fluorogenic prodrug **1a** and its stable control **1b** via the seven-step synthetic protocol described in the Supplement. These compounds are soluble at least up to 30 μM in phosphate buffer (pH 7), DMSO (1%, v/v) mixtures. Higher concentrations were not tested.

### 4.2 Mice

Wild type (WT) BALB/c.Q mice and BALB/c.Q mice with a mutation in the *Ncf1* gene (Ncf^m1j/m1j^, denoted as *Ncf1∗∗*), which affects the Ncf1 (p47phox) protein's ability to translocate to the membrane upon activation and therefore blocks the NOX2 complex's ability to function [39,40] were bred and maintained at the animal facilities of the University of Erlangen, Germany and Lviv, Ukraine. Mice were kept on 12 h light/dark cycles and had free access to standard rodent chow and water. Experiments were performed on both female and male littermates between 8 and 16 weeks of age, frequency-matched for sex and age, and evaluated with blinded identity. Mice were allocated randomly into groups, such that each cage contained animals of every group to compensate for possible cage effects. Sample sizes were chosen to ensure adequate power according to estimates of the effect size strength, on the basis of previous experience with the MSU injection model. All procedures were in accordance with institutional guidelines on animal welfare and were approved by the local ethical committee of the university Erlangen-Nürnberg (permit numbers RUF 55.2.2-2532.2-1041-15 and TS-12/15 Medizin III Klin Im.), Lviv (permits 20201221/9 and 20230831/8), and ICBP-NS (permit ANSVSA 20250930/20).

### 4.3 Human subjects

We used blood from 3 patients with chronic granulomatous disease (CGD) during this study and age- and sex-matched normal healthy donors (NHDs). Diagnosis of CGD was made both clinically and by molecular genetic analysis. Patient 1 (29 years old, male) showed compound-heterozygous mutations and a typical clinical and granulocyte phenotype, patient 2 (49 years old, male) had an X-linked CGD with a frameshift mutation in the CYBB gene, and patient 3 (35 years old, male) carried two mutations in the CYBA gene. All experiments were approved by the ethical committee of the university of Erlangen (permits number Re.-No.3982 and 193_13B). All subjects included in the study gave written informed consent.

### 4.4 MSU crystal-induced arthritis

Monosodium urate (MSU) crystals were prepared in house as previously described [12]. To induce monoarthritis, 70 μl of a 20 mg/ml suspension of MSU crystals in PBS was injected subcutaneously into the left hind paw, between the metatarsals. Measurement of paw thickness was then conducted using an electronic caliper. Paw images were captured on a cell phone equipped with an S5KGW1 photo sensor set to an exposure period of 1/100s ISO 800.

### 4.5 Histological imaging of MSU crystal-induced arthritis

For histomorphological analysis, paws from prodrug 1- or vehicle-treated *Ncf1∗∗* mice 11 days after injections of MSU crystals were fixed overnight in 4% formalin, decalcified with EDTA, and embedded in paraffin. Paraffin sections were then stained with hematoxylin & eosin or tartrate-resistant acid phosphatase (TRAP) for assessment of inflammation and osteoclasts, respectively.

### 4.6 Microcomputed tomography (μCT)

Prodrug 1- or vehicle (intralipid) treated *Ncf1∗∗* mice were euthanized 10-14 days after induction of MSU crystal-induced arthritis. Specimens were prepared by separating the paws from the surrounding soft tissue. The isolated paws were then stored in 70% ethanol until the time of measurement. μCT of the metatarsal and tarsal areas of these mice was performed using the cone-beam desktop microcomputer tomograph "μCT40" from SCANCO Medical AG, Bruettisellen, Switzerland. The settings were optimized for calcified tissue visualization at 55 kVp, 145 μA, and 200 ms integration time.

### 4.7 Airpouch model

Mice were administered 50 μl of 25 mM prodrug **1** or vehicle subcutaneously on a daily basis until one day before euthanasia. The treatment was initiated on the day of the first air injection to establish the air pouch. To create air pouches, isoflurane-anesthetized mice were injected s.c. with 3 ml of sterile air. On the third day after the initial injection, an additional 2 ml of sterile air was injected into the preexisting pouch. On the following day, an injection was performed by introducing 5 mg MSU crystals in 500 μl PBS into the air pouch. After 24 h, the mouse was euthanized and a lavage of the air pouch was conducted using 500 μl PBS. Cytokine and chemokine concentrations in the lavages were measured by multiplex bead technology (LegendPlex) and quantified by cytofluorometry on a Gallios flow cytometer.

### 4.8 Treatment with prodrug 1 *in vivo*

50 μl of a 25 mM solution of prodrug **1** (1,25 μmol) in Intralipid-20% (Sigma) was administered to mice via subcutaneous injections once daily at 24-h at the back skin. For each injection, a fresh batch of injection solution was prepared and dissolved using an ultrasonic bath. In the MSU crystal-induced arthritis model, treatment was initiated 2 days prior to MSU crystal injection and administered daily for the rest of the experiment or withdrawn 4 days after MSU crystal injection. The airpouch model treatment was initiated on the same day as the establishment of the air pouch and was sustained for the subsequent 4 days until the day anteceding lavaging of the air pouch.

### 4.9 Isolation of neutrophils from human peripheral blood

Neutrophils were freshly isolated from human whole blood obtained by venipuncture from healthy volunteer donors or individuals with CGD who had provided written informed consent. The blood was anticoagulated with EDTA and placed on Histopaque1077 for density gradient separation. Samples were centrifuged at 1400 rpm for 30 min at room temperature with low acceleration and without centrifuge brakes. Suspension above the buffy coat was removed, and the white layer containing the polymorphonuclear neutrophils (PMNs) on the top of the erythrocytes was collected. To remove contaminating erythrocytes, PMNs were subjected to short cycles of hypotonic lysis with deionized water and washing with PBS. After restitution of normal osmolality with R0, viable cells were counted on the “Luna” Cell Counter (Labtech). Cell suspension purity of CD45^+^CD16^+^CD14^−^ PMNs was determined using flow cytometry, and routinely exceeded 95%. The cells were then adjusted to a concentration of 1 × 10^6^ cells/ml or 1 × 10^8^ in serum-free RPMI 1640 (R0). All analyses of human blood samples were performed in accordance to the institutional guidelines and with the approval of the Ethical Committee of the University Hospital Erlangen (Permit 193_13B).

### 4.10 Isolation of murine neutrophils from blood and bone-marrow

Blood was collected from mice using the submandibular vein and transferred into an EDTA tube. The blood was then subjected to two rounds of hypotonic erythrocyte lysis and subsequently washed with PBS. The cell concentration was enriched to 1 × 10^6^ cells/ml using the negative selection Stem Cell Neutrophil Enrichment Kit following the manufacturer's protocol. Alternatively, neutrophils were isolated from whole mouse blood using the Miltenyi MACSxpress Whole Blood Neutrophil Isolation Kit according to the manufacturer's instructions.

Bone marrow isolation was conducted by removing excess soft tissue from the bone surface of the tibia and femur using a scalpel and forceps, while avoiding harsh scraping of the bone surface. The femurs and tibias were cleaned and subsequently immersed in 70% EtOH for 30 s. They were then transferred to a solution of PBS for storage. A 10 ml syringe was attached to a 21G needle and used to draw fresh PBS. The bones were retrieved using forceps and subsequently, the epiphyses were removed. The bone marrow extraction procedure involved inserting a needle into one end of the hollow bone, followed by a gentle flushing out of the entire bone marrow contents. The solution obtained was subjected to clearance using a 70 μm cell strainer. A single cell suspension was obtained by pipetting the bone marrow aspirate. Isolation of leukocytes was performed using histopaque gradient centrifugation. A gradient was prepared by layering 4 ml of histopaque 1077 onto 4 ml of histopaque 1119. The layered solution underwent centrifugation at 800*g* for 40 min with low acceleration and no brakes. After centrifugation, the lower band of the two was collected. Purity was determined by antibodies to Ly6C, CD11b and Ly6G and was routinely above 95%.

### 4.11 Measurement of reactive oxygen species in human and mouse blood

For measuring intracellular ROS in blood, blood was collected into EDTA tubes and erythrocytes were lysed by hypotonic lysis. (9 ml cold deionized water, 20s; 1 ml 10xPBS, mix gently – Centrifuge 1200 rpm, 7min. Resuspend pellet 1 ml PBS). White blood cells were loaded with 5 μM CM-H2DCFDA or mitoSOX for 30 min in R0. Afterwards, they were treated with various doses of pro-NAAFs or negative controls (ferrocene or 1% DMSO) for 2 h. The positive controls PMA (100 ng/ml) and A23187 (1 μM) were only added for 15 min. Cells were then incubated with antibodies to CD11b, Ly6C, CD3, and CD19 for 12 min on ice. After washing with PBS, cells were centrifuged at 1200 rpm for 7 min. The resulting pellet was resuspended in 300 μl of FACS Buffer and analyzed using a Beckman Coulter Gallios flow cytometer.

### 4.12 Plate reader assay for measurement of extracellular DNA extrusion

150,000 cells/well isolated human or murine neutrophils were stimulated in 96-well flat bottom plates for 4 h in R0 medium with various concentrations of pro-NAAFs, 100 ng/ml PMA, or the calcium ionophore A23187 (1 μM). Vehicle (1,5% DMSO) or Ferrocene was used as a negative control. To detect extracellular DNA, SytoxGreen, a cell-impermeable dye, was used at a concentration of 2.5 μM. The measurement (excitation 485 nm, emission at 535 nm) was done on an Infinite® 200 PRO TECAN plate reader at 37°C, 5% CO_2_.

### 4.13 Microscopical imaging of neutrophil extracellular traps

Cells were incubated with various concentrations of pro-NAAFs, 100 ng/ml PMA, and 1 μM A23187 in R0 at 37°C/5%CO_2_, following the same procedure as for measurement of extracellular DNA extrusion. Cells were then fixed with 4% paraformaldehyde (PFA) for 20 min. After fixation, cells were permeabilized with Triton X-100 for 5 min and washed three times with phosphate-buffered saline (PBS). The cells were then incubated with blocking buffer for 1 h at room temperature, followed by incubation with primary antibodies to neutrophil elastase in blocking buffer overnight at 4°C. After that, they were washed three times with PBS and subsequently incubated with secondary antibody and nuclear staining with Hoechst for 1.5 h at room temperature. After washing two times with PBS as well as with water and sample were mounted using either Mounting Medium or Mowiol. The samples were then examined under a Keyence microscope.

### 4.14 Aggregated NET formation and cytokine degradation

Neutrophils were isolated from whole human blood using Histopaque1077 at a density of 1 × 10^8^ cells/ml in R0. Aggregated NETs (aggNETs) were formed from isolated neutrophils as previously described [12,13] by stimulation with prodrug **1**, 100 ng/ml PMA, and 20 pg/cell MSU crystals for 4 h in an incubator at 37°C and 5% CO_2_. The aggNET suspensions were then passed through a 40 μm strainer to remove cell culture supernatants and excess intact cells. The purified aggNETs were washed three times with PBS and incubated in PBS with a mixture of recombinant cytokines and chemokines for 18 h at 37°C/5%CO_2_. Remaining cytokines and chemokines were quantifiied using a LegendPlex Panel by flow cytometry on a Gallios flow cytometer.

### 4.15 Detection of inflammasome activation

Isolated neutrophils from whole blood of healthy human volunteers (2 males and 2 females, aged 25–38 years) were stimulated with 100 ng/ml lipopolysaccharide for 90 min at 37°C in a humidified incubator. Subsequently, monosodium urate crystals were added at a concentration of 200 μg/ml and incubated for 15 min. Vehicle (DMSO) or 100 μM prodrug **1** was then added to the wells, followed by an additional 30 min-incubation. Inflammasome activation was assessed using the FAM-FLICA™ Caspase-1 Assay Kit (YVAD-FMK 660). Cells were stained according to the manufacturer's instructions. The FLICA reagent was added directly to the cell culture medium during the final 30 min of incubation. After incubation, the plates were centrifuged at 300 × g for 10 min at room temperature. Cells were washed with PBS, fixed, and analyzed on an Cytoflex S flow cytometer (Beckman Coulter). IL-1β concentrations in neutrophil culture supernatants were quantified using the ELISA MAX™ Deluxe Set Human IL-1β (BioLegend).

### 4.16 Measurement of markers of oxidative stress

Blood samples were obtained from mice prior to the initiation of Prodrug 1 treatment and arthritis and at endpoint. The blood was collected in a plasma centrifugation tube and centrifuged at 15,000 rpm for 1.5 min. The collected supernatant was then frozen at −20°C for preservation. Carbonylated proteins in the serum were measured using a kit. Additionally, oxysterol extraction and quantitation were performed as previously described [68]. Plasma was resuspended in lysis buffer (70 μl 0.1% Triton X 100, 40 μl DMSO, 5 μl BHT at 90 mg ml^−1^) and sonicated for 20 min. Standards and quality controls were prepared by spiking 30 μl plasma with a concentration series of nine authentic oxysterols (0 to1 ng μl^−1^). An external standard, 22SOHC D7 (3 μl, 50 ng μl^−1^), was added to all samples and standards and vortexed. Lipid extraction was performed by adding 190 μl LC MS–grade methanol and 380 μl dichloromethane (DCM), followed by 20 s of vortexing. LC MS–grade water (120 μl) was added, vortexed for 10 s, and samples were left at room temperature for 10 min. Samples were centrifuged at 7168 g, 8°C for 10 min. The lower DCM layer (∼350 μl) was collected and dried under N_2_ using a Turbovap LV (Biotage). A 15 μl aliquot of the lipid extract was dried separately for cholesterol quantification using the Invitrogen™ Molecular Probes™ Amplex™ Red Cholesterol Assay Kit (10236962).

The dried DCM extract was reconstituted in 500 μl methanol and 1.5 ml LC MS grade water with 0.1% formic acid. Oxysterols were isolated using Oasis HLB SPE cartridges (Waters), preconditioned with 800 μl methanol and 600 μl water + 0.1% formic acid. Samples were loaded, and flow through discarded. Cartridges were washed with 600 μl water +0.1% formic acid and 600 μl hexane. Oxysterols were eluted with 1 ml butyl acetate, dried under N_2_, and reconstituted in 40 μl 40% methanol +0.1% formic acid for LC MS/MS.

Standards (10 μl) and samples (20 μl) were injected onto a NUCLEOSIL C18 column (100 5125/2) with a guard column. Solvent A was 70% methanol, 10% water, 0.1% formic acid; solvent B was 90% isopropanol, 10% methanol, 0.1% formic acid. A multistep gradient was applied on an ACQUITY UPLC system (Waters): 0-7 min, 16% B; 7-11 min, 16–24% B; 11-25 min, 24-100% B; 25-30 min, 100% B; 30-32 min, 100-16% B; held at 16% B until 48 min.

Mass spectrometry (MS) was performed on a Xevo TQ S Triple Quadrupole (Waters) in positive ESI mode. Nitrogen was used for desolvation (500°C, 900 L h^−1^) and argon for collision (0.15 ml min^−1^). MRM transitions were optimized using authentic standards (Avanti Polar Lipids): 24(S) hydroxycholesterol, 25 hydroxycholesterol, 27 hydroxycholesterol, 7α hydroxycholesterol, 7 ketocholesterol, 7α,27 dihydroxycholesterol, 7α,24(R/S) dihydroxy-cholesterol, and 7α,25 dihydroxycholesterol. Each batch included an unprocessed standard mixture and pooled samples as quality controls.

MS data were processed using MassLynx and TargetLynx (Waters). Oxysterols were identified by matching retention time, exact mass, and MS/MS spectra to authentic standards. Extraction efficiency and recovery were assessed by spiking authentic standards into plasma prior to extraction and LC MS/MS analysis. Peak detection and integration were performed using the ApexTrack algorithm in MassLynx, which automatically determines peak width and threshold parameters. Peak areas for the quantifier MRM transitions were extracted for each analyte. Standard curves were generated using authentic oxysterols at 0, 0.005, 0.010, 0.025, 0.050, 0.075, 0.1, 0.25, 0.5, 0.75, and 1 ng μl^−1^. Linear regression was used to derive the best fit equation, and sample concentrations were calculated accordingly.

Protein carbonylation was measured in mouse samples by the Protein Carbonyl Content Assay Kit (Sigma).

### 4.17 IR imaging

*In vivo* imaging was performed using a Li-COR Pearl Trilogy Imager (LI-COR Biosciences GmbH, Germany). Excitation was performed with the 685 nm laser and emission was analyzed at the 720 channel, respectively, using a 85 μm resolution. Images were normalized using native Image Studio software, provided by the device manufacturer and all measurement parameters were strictly controlled to be identical within the measurement series, as represented in each image. Paw areas were selected and signal intensity in the MSU crystal-injected versus NaCl-injected paw was then determined.

### 4.18 Prodrug 1 treatment and tissue boron quantification

WT and *Ncf1∗∗* mice were injected subcutaneously (s.c.) with Prodrug **1** once daily for 5 consecutive days. On day 3, monosodium urate crystals were injected subcutaneously into the left hind paw, between the metatarsals, to induce acute inflammation. On day 5, mice were euthanized and organs were harvested. Tissues were mechanically dissociated using a gentleMACS™ dissociator (Miltenyi) to generate homogenized tissue suspensions, and boron concentrations were determined using a colorimetric assay according to the manufacturer's instructions.

### 4.19 Luminol and isoluminol measurement

Neutrophils were isolated from whole blood of human healthy donors by density gradient centrifugation. Following isolation, cells were resuspended in in RPMI 1640 medium containing glucose (1 mg/ml), HEPES (25 mM), and 20 μg/ml luminol or isoluminol and treated with various concentrations of prodrug 1, 100 ng/m PMA, 1 μM A23187 or vehicle (DMSO equivalent to the amount of DMSO present upon treatment with 100 μM prodrug 1). For some experiments NOX2 was inhibited by 15 min preincubation with 10 μM GSK2795039 (Sigma-Aldrich). For luminescence measurements, cells were immediately seeded after activation into 96 well plates and chemiluminescence associated with total (luminol) or released (isoluminol) ROS was measured at 37°C for up to 60 min using a Tecan microplate reader.

### 4.20 RNA isolation for bulk RNA sequencing

Paws were dissected to remove skin and tendons, then immediately placed in RNA later for stabilization and stored at −20°C. The paws were briefly dried upon thawing, and 1 ml of Trizol was added to each sample. The tissues were homogenized, and the homogenates were centrifuged at 4°C to collect the supernatants. Chloroform was added and mixed by shaking, and the samples were incubated for 5 min at room temperature. Following centrifugation, the upper phases were carefully transferred to new tubes and mixed with ethanol, and RNA purification was completed using the Qiagen RNeasy Mini Kit, including DNase I digestion.

### 4.21 Single-cell RNA-Seq analysis pipeline

Raw gene expression matrices were generated for each sample by a custom pipeline combining ‘kallisto’ (v.0.48.0) and ‘bustools’ (v.0.46.1) using GRCm38 as mouse reference. The output-filtered gene expression matrices were analyzed in ‘R' (v.4.2.1), where empty droplets and doublets were removed for each sample using the packages 'DropletUtils' (v.1.8.0) and ‘doubletFinder’ [69], and further analysis was performed using the ‘Seurat’ (v.4.3) package. Subsequently, cells were detected by ranking cell barcodes according to their number of unique molecular identifiers (UMIs) captured using the barcodeRanks function. Low-ranked cells from this process were labelled as false positives and were discarded, yielding 81x106 unique reads with an average of 7000 reads per cell. The criteria for filtering the dataset were as follows: (i) genes expressed in more than three cells; (ii) cells expressing more than 200 genes; (iii) low-quality cells were removed if they included more than 25% UMIs from the mitochondrial genome. Mitochondrial content can vary between humans and mice based on scRNA-seq technology. We set the filter to 25% after calculating mitochondrial content for each sample. Gene expression matrices were normalized by the NormalizeData function, and 3000 features with high cell-to-cell variation were calculated using the FindVariableFeatures function. We identified ‘anchors’ between individual datasets with the FindIntegrationAnchors function for batch correction of gene expression of all cells across sequencing datasets. Datasets with multiple samples may be affected by batch effects, which should be addressed using data integration methods. In this study, data integration was performed using the IntegrateData function with Canonical Correlation Analysis (CCA), specifying 30 dimensions, to produce a batch-corrected integrated assay. The dimensionality of the dataset was reduced to 100 principal components of the linearly scaled data using the ScaleData and RunPCA functions, respectively. Finally, we clustered cells using the FindNeighbors and FindClusters functions and performed nonlinear dimensionality reduction by uniform manifold approximation and projection for dimension reduction (UMAP) with the RunUMAP function, using 30 dimensions for all approaches. The FindAllMarkers function in Seurat was used to find markers for each unique cluster. Clusters were identified and annotated based on expression of canonical markers. The neutrophils were subseted based on their expression of Ly6G. Subsequently, for clustering the neutrophils, the previous described workflow was repeated.

### 4.22 Determination of cell abundance

We used the ‘scProportion Test’ from the R library scProportionTest’ to quantify differences in cell abundance between clusters from scRNA-seq samples. A permutation test was used to calculate a statistical p-value for each cluster, and a confidence interval for the magnitude difference was returned via bootstrapping.

### 4.23 Differential gene expression and gene set enrichment

Differential gene expression analysis of the single-cell data was performed per cell cluster and PCS group using the MAST method (Model-based Analysis of Single-cell Transcriptomics), as implemented in the FindMarkers function from the Seurat R package. MAST uses a hurdle model tailored for single-cell RNA-seq data, allowing it to model both the discrete and continuous components of gene expression and incorporate sample-level covariates to reduce pseudoreplication bias [70]. Benchmarking analyses revealed that MAST ranked among the best-performing methods under conditions of moderate sequencing depth and batch effects, which closely reflected the characteristics of our dataset. Gene set enrichment was calculated using a non-parametric Wilcoxon Mann-Whitney test on the log fold changes between the cell groups as implemented in R library gage (v. 2.52, [71]).

### 4.24 RNA velocity analysis

Transcriptional dynamics were modeled using scVelo (v 0.2.5) to infer RNA velocity from spliced and unspliced mRNA abundances. After filtering for 3000 highly variable genes and computing moments, velocities were estimated using the stochastic model. To visualize directed transitions between cell states, velocity vectors were projected onto the UMAP embedding.

### 4.25 Gene-set permutation analysis of ROS regulator expression

Human cytosolic and mitochondrial ROS regulator genes were taken from Bennet et al. [45]. Human genes were mapped to mouse orthologs, and only orthologs present in the corresponding mouse expression dataset were analyzed.

Bulk RNA-seq expression values from paws from vehicle- or prodrug 1-treated *Ncf1∗∗* mice at day 2 and day 14 were analyzed. For each gene set and time point, the observed statistic was the mean Δlog_2_ expression across all matched mouse genes. Significance was assessed by 50,000 permutations using random gene sets of identical size drawn from the expression background. Genes belonging to the combined list of mitochondrial and cytosolic ROS-regulators were excluded from the random background to avoid sampling functionally related ROS regulators into the null sets. The empirical null distribution consisted of mean Δlog2 values from the random gene sets. Two-sided and one-sided empirical p-values were calculated from this distribution to test for overall deviation and directional enrichment toward prodrug 1 or vehicle, respectively. A permutation z-score was calculated as: z = (T_observed_−μ_null)_/σ_null_, where positive z-scores indicate prodrug 1-high enrichment and negative z-scores indicate vehicle-high enrichment. Gene sets with p < 0.05 were considered significantly shifted.

### 4.26 4 color death stain

For subclassification of neutrophil cell death phenotypes we employed a flow cytometric 4-color staining technique including annexin A5-FITC, propidium iodide, DiIC1(5), and Hoechst 33342 as previously described [37]. In brief, neutrophil isolated from human peripheral blood were incubated with different concentration of Prodrug **1**, **2** or **3** or DMSO (vehicle). After washing, neutrophils were stained with 1 μg/ml Annexin V-FITC (Biozym; for detecting phosphatidyl serine present on apoptotic cells), 20 μg/ml propidium iodide (Sigma; for measuring cell membrane damage), 10 nM DilCl(5) (1′,3, 3, 3′, 3′ -Hexamethylindodicarbo-cyanine iodide; Invitrogen; for quantifying mitochondrial membrane potential), and 1 μg/ml Hoechst33342 (Invitrogen; for staining DNA) for 15 min and then analyzed on a Beckman Coulter Gallios flow cytometer.

### 4.27 Statistical analysis

Two group comparisons were performed using unpaired or paired 2-tailed Student's *t*-test or, in the case of non-normally distributed data, by 2-tailed Mann-Whitney *U* test. Gaussian distribution of samples was checked using D'Agostino-Pearson omnibus normality test. For comparing the mean of every group to a fixed value we used a One sample *t*-test. Within each set of experiments shown in one graph multiple comparisons of groups were performed by ANOVA and adjusted using Sidak's (if the means of selected pairs of groups were compared) multiple comparisons test, or Dunnett's test if several means were compared to a control mean. In case of non-normally distributed data we used Kruskal-Wallis or Friedman's test with Dunn's post hoc test for multiple comparisons. Adjusted p values < 0.05 were considered statistically significant. Computations and charts were performed using GraphPad Prism 11 software. Levels of significance are allocated as follows throughout the whole manuscript: ns, not significant; ∗p < 0.05; ∗∗p < 0.01; ∗∗∗p < 0.001. Unless indicated otherwise, figures show means ± SEM from different biological replicates.

## Declaration of the use of generative AI tools

During manuscript preparation the authors used OpenAI ChatGPT (Version 5.5) for literature search figure reformatting, and for aligning the manuscript to the formatting requirements of the journal. After using this tool, the authors reviewed and edited the content as needed and take full responsibility for the content of the published article.

## CRediT authorship contribution statement

**M. Euler:** Data curation, Formal analysis, Investigation, Methodology, Visualization, Writing – original draft, Writing – review & editing. **O. Hattab:** Data curation, Formal analysis, Investigation, Writing – review & editing. **K. Borah Slater:** Formal analysis, Investigation, Methodology, Writing – review & editing. **B. Golub:** Methodology, Resources, Writing – review & editing. **R. Selin:** Methodology, Resources, Writing – review & editing. **Z. Cheng:** Investigation, Writing – review & editing. **Y.A. Maluje Villanueva:** Data curation, Formal analysis, Visualization, Writing – review & editing. **G.H. Özkan:** Methodology, Resources, Writing – review & editing. **G. Bila:** Investigation, Writing – review & editing. **K. Dutta:** Investigation, Writing – review & editing. **D. Weidner:** Formal analysis, Investigation, Writing – review & editing. **E.J. Hoffmann:** Investigation, Writing – review & editing. **J. Friscic:** Supervision, Writing – review & editing. **C.D. Sadik:** Supervision, Writing – review & editing. **T. Harrer:** Resources, Writing – review & editing. **J. Köhl:** Formal analysis, Resources, Writing – review & editing. **G. Schett:** Conceptualization, Funding acquisition, Resources, Writing – review & editing. **L. Munoz:** Resources, Supervision, Writing – review & editing. **A. Fähnrich:** Data curation, Supervision, Visualization, Writing – review & editing. **S. Murthy:** Formal analysis, Writing – review & editing. **R. Grieshaber-Bouyer:** Data curation, Formal analysis, Writing – original draft, Writing – review & editing. **M. Herrmann:** Conceptualization, Funding acquisition, Supervision, Writing – review & editing. **R. Bilyy:** Formal analysis, Funding acquisition, Methodology, Resources, Supervision, Writing – review & editing. **H.R. Griffiths:** Data curation, Formal analysis, Funding acquisition, Supervision, Writing – review & editing. **A. Mokhir:** Conceptualization, Formal analysis, Funding acquisition, Supervision, Writing – original draft, Writing – review & editing. **M.H. Hoffmann:** Conceptualization, Formal analysis, Funding acquisition, Supervision, Visualization, Writing – original draft, Writing – review & editing.

## Declaration of competing interest

The authors declare the following financial interests/personal relationships which may be considered as potential competing interests:Markus H Hoffmann reports financial support was provided by German Research Foundation. Martin Herrmann reports financial support was provided by German Research Foundation. Markus H Hoffmann reports financial support was provided by European Commission. Andriy Mokhir reports financial support was provided by European Commission. Rostyslav Bilyy reports financial support was provided by European Commission. Helen R Griffiths reports financial support was provided by European Commission. Rostyslav Bilyy reports financial support was provided by National Research Foundation of Ukraine. If there are other authors, they declare that they have no known competing financial interests or personal relationships that could have appeared to influence the work reported in this paper.

## Data Availability

Data will be made available on request.
