# Supplementary material for: NADPH oxidase 2 (NOX2)-independent inducers of neutrophil extracellular traps promote resolution of chronic inflammation
Source: Redox Biol. 2026 Jul 15;95:104302. doi: 10.1016/j.redox.2026.104302 (PMC13393013; doi:10.1016/j.redox.2026.104302)
Supplement: Multimedia component 1 [file mmc1.docx]

**SUPPLEMENTARY FIGURES**

**
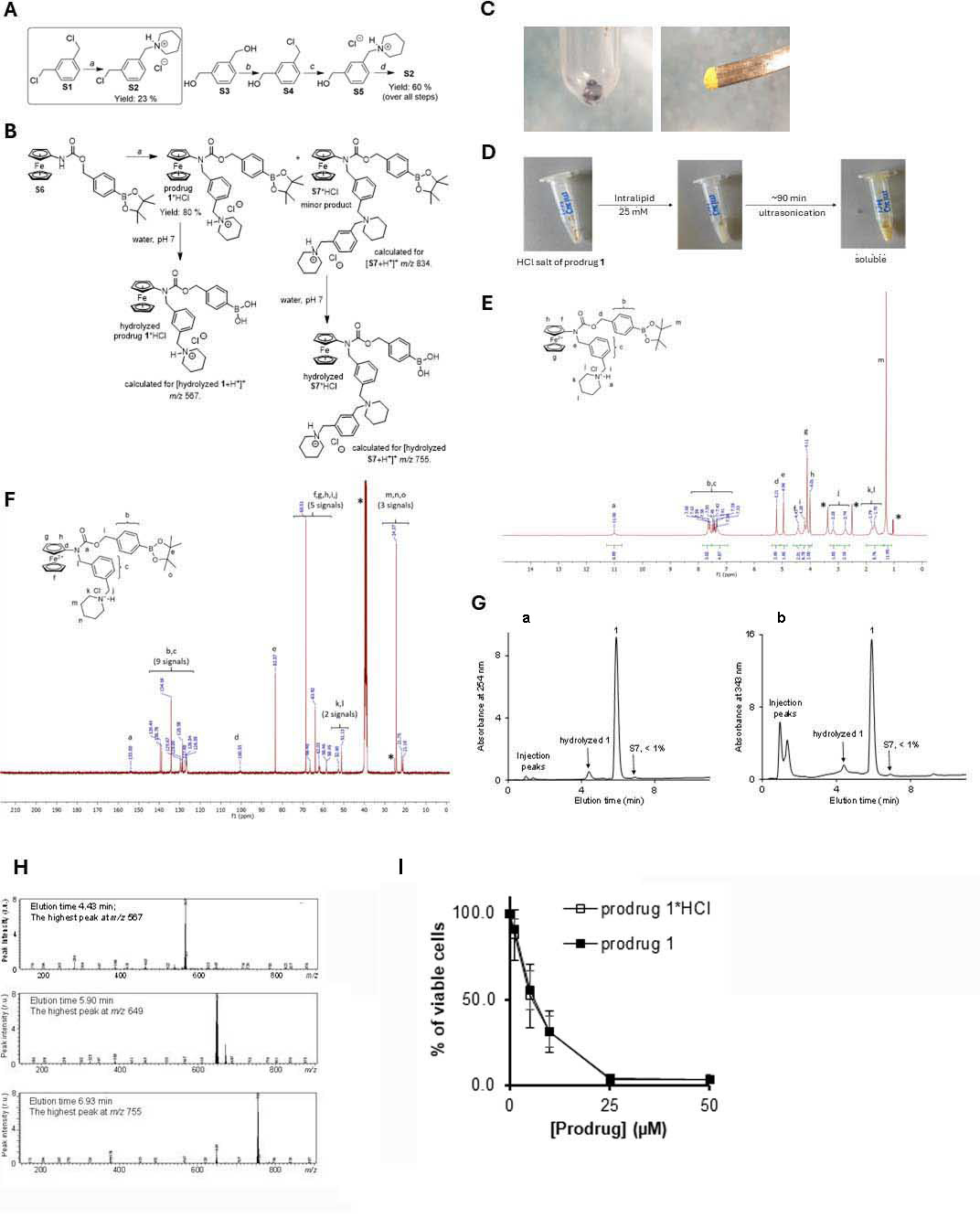
**

**Figure S1. (A)** Improved synthesis of intermediate S2. Insert: previously reported synthesis of S2. **(B)** Improved synthesis of prodrug **1**. In aqueous solution prodrug **1** and the minor product S7 are partially hydrolyzed. **(C)** Left image: Appearance of prodrug 1 prepared by using the previously reported protocol; right image: Appearance of HCl salt of prodrug **1** prepared by using the optimized protocol. **(D)** Solubilization of prodrug **1** in Intralipid-20% (the final mixture appears milky). **(E)** ^1^H NMR spectrum of HCl salt of prodrug **1**. Peaks of residual DMSO-d5 in DMSO-d6, water and the impurity at ~1 ppm are indicated with *. **(F)** ^13^C NMR spectrum of HCl salt of prodrug **1**. Peaks of DMSO-d6 and the impurity at ~24 ppm are indicated with *. **(G)** HPLC analysis of prodrug **1***HCl. Conditions: HPLC column - Macherey-Nagel, EC, NUCLEODUR C18 HTec, 5 µm, 50x2 mm; gradient: in 11 min from 15 to 95 % eluent B in A, for 4.5 min at 95 % B, for 2.5 min at 15 % B. Eluent a: ammonium formate (10 mM) with formic acid (FA, 0.1%, v/v); eluent b: acetonitrile with FA (0.1%, v/v). **(H)** Electrospray ionization (ESI) mass spectra of three fractions (at 4.43, 5.90 and 6.93 min) from the HPLC analysis shown in (G). The calculated *m/z* values for prodrug **1**, its hydrolyzed form as well as side product S7 and its hydrolyzed form are provided in (B). **(I)** Effects of prodrug **1** in the form of salt (prodrug **1***HCl obtained using the protocol described in (B) and free base (prodrug **1**, obtained as previously described [53]) on the viability of the human ovarian cancer A2780 cell line. The percentage of viable cells was determined by using an MTT assay as previously described [53].


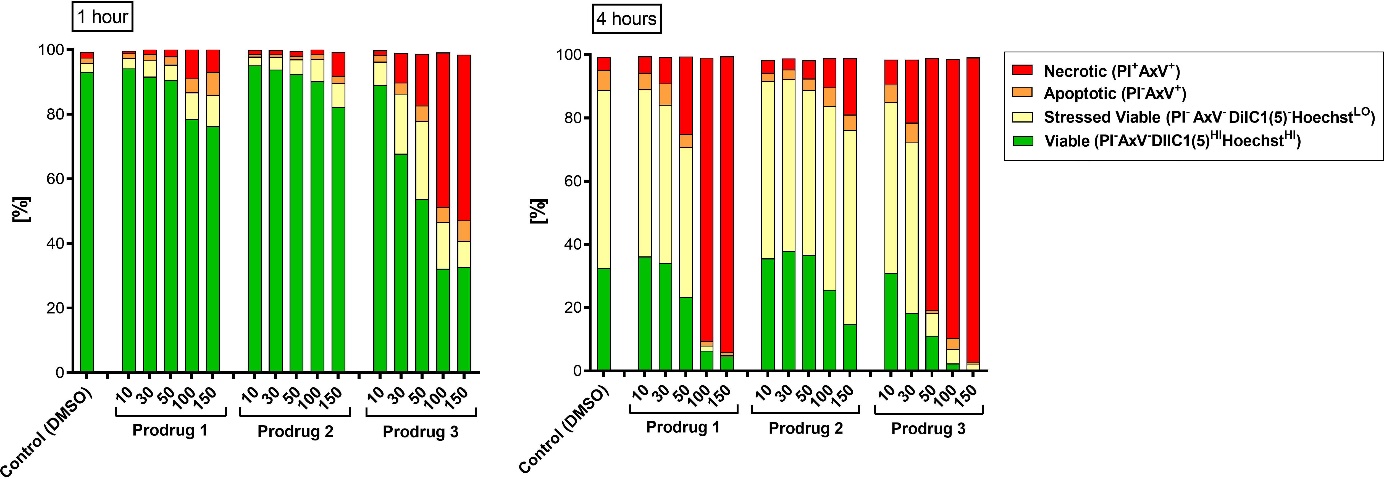


**Figure S2. Types of cell death induced by pro-NAAFs.** Flow cytometric analysis [31] of percentages of necrotic, apoptotic, stressed viable and viable human blood-derived neutrophils after 1 hour or 4 hour incubation with pro-NAAFs. Plots show mean percentages of cells from 3 normal healthy blood donors.

**
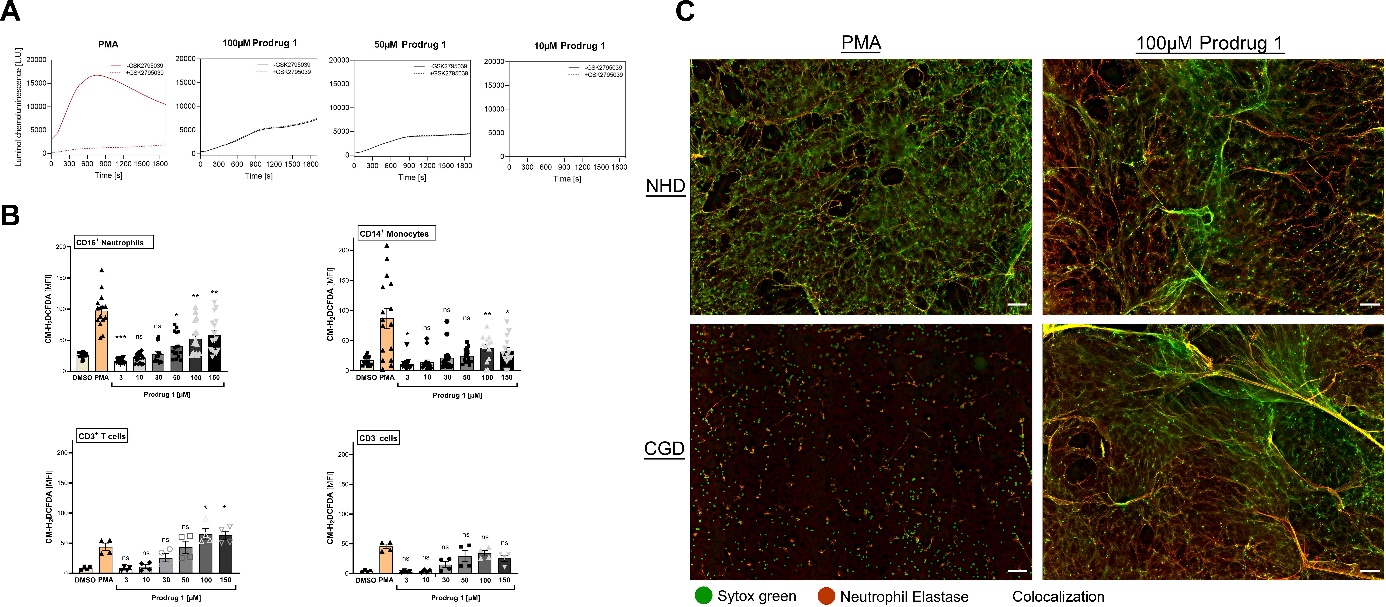
**

**Figure S3. ROS induction and NET formation in response to Prodrug 1 in human cells.** **(A)** Dynamics of total ROS formation in human blood-derived neutrophils upon pre-incubation with the NOX2-inhibitor GSK2795039, as determined by luminol luminescence. Curves show mean results from 3 normal healthy donors (NHDs). **(B)** Absolute levels of Intracellular ROS accumulation in cells from NHD peripheral blood. Results from cells of 4-16 donors. *p < 0.05, **p < 0.01, ***p < 0.001, ns, not significant, as compared to DMSO control. ANOVA with Dunnet´s multiple comparisons test. MFI, mean fluorescence intensity. **(C)** Representative fluorescence microscopy images from neutrophils isolated from peripheral blood of human NHD or individuals with CGD. Scale bars, 100 µm.


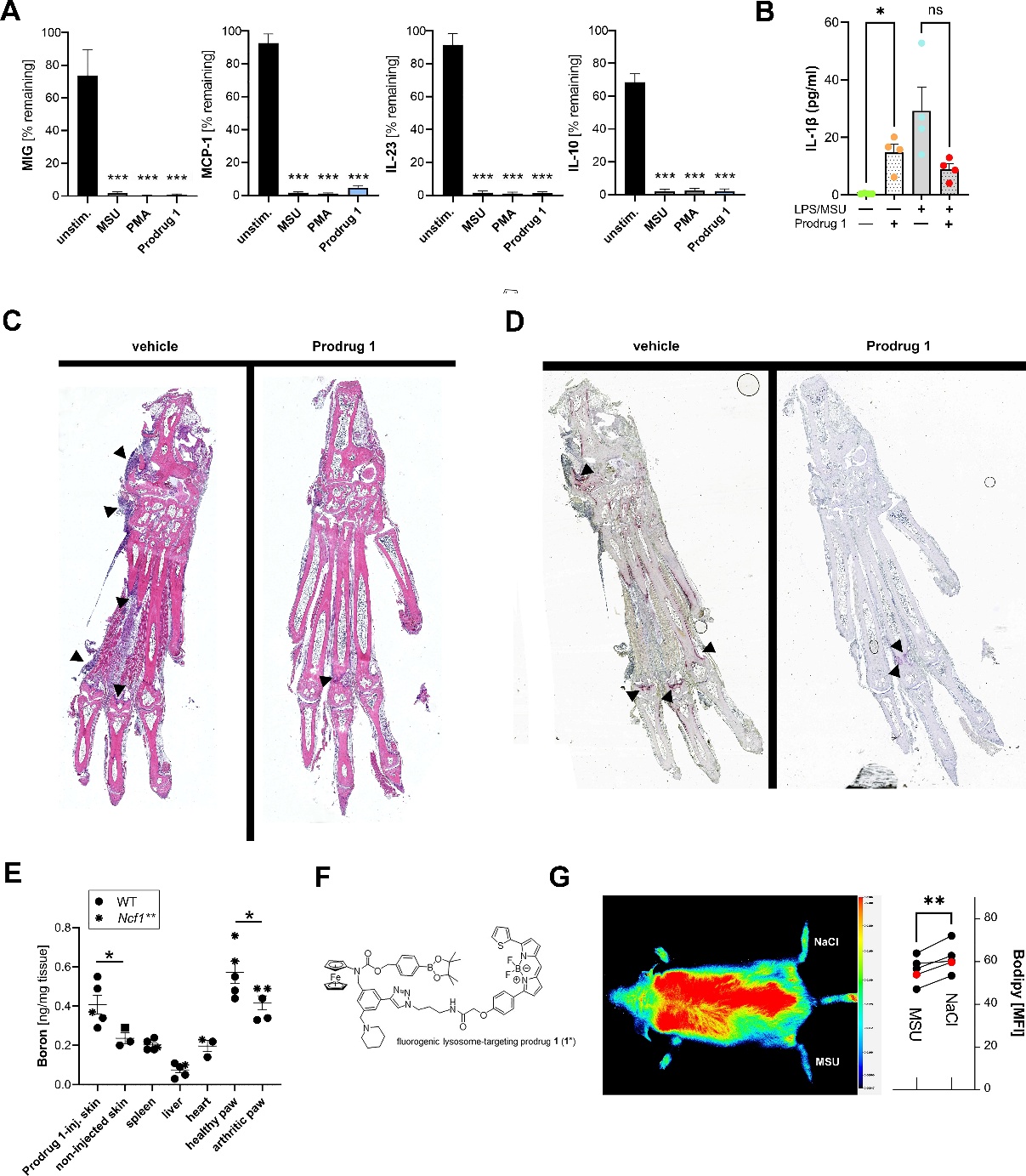


**Figure S4. (A)** Degradation of inflammatory mediators by prodrug 1-induced aggregated NETs. ***p < 0.001, as determined by ANOVA with Dunnett´s multiple comparisons test. **(B)** Interleukin-β concentrations in supernatants from human blood neutrophils after stimulation with lipopolysaccharide (LPS) and MSU crystals and co-incubation with prodrug **1**. N = 4 normal healthy blood donors/condition. *p < 0.05, n.s., not significant, paired ANOVA with Sidak´s multiple comparisons test. **(C, D)** Inflammatory and bone changes in paws of vehicle- or prodrug **1**-treated mice with Monosodium urate (MSU) crystal-induced arthritis. (B) H&E staining of sections from *Ncf1*** mouse paws 11 days after injection of MSU crystals. Black arrowheads indicate inflammatory infiltrates. (C) Tartrate acid-resistant phosphatase (TRAP) staining of sections from *Ncf1*** mouse paws 11 days after injection of MSU crystals. Black arrowheads indicate bone-resorbing osteoclasts. **(E)** Boron concentrations in extracts from different mouse organs after MSU crystal-induced arthritis and subcutaneous treatment with prodrug **1**. *p < 0.05, Student´s t-test. **(F)** Chemical structure of the fluorogenic version of prodrug **1**. **(G)** Image and quantification of uptake of Bodipy-labelled prodrug **1** into paws. ** p < 0.01, paired Student´s t-test. The according values to the representative image are shown in red.


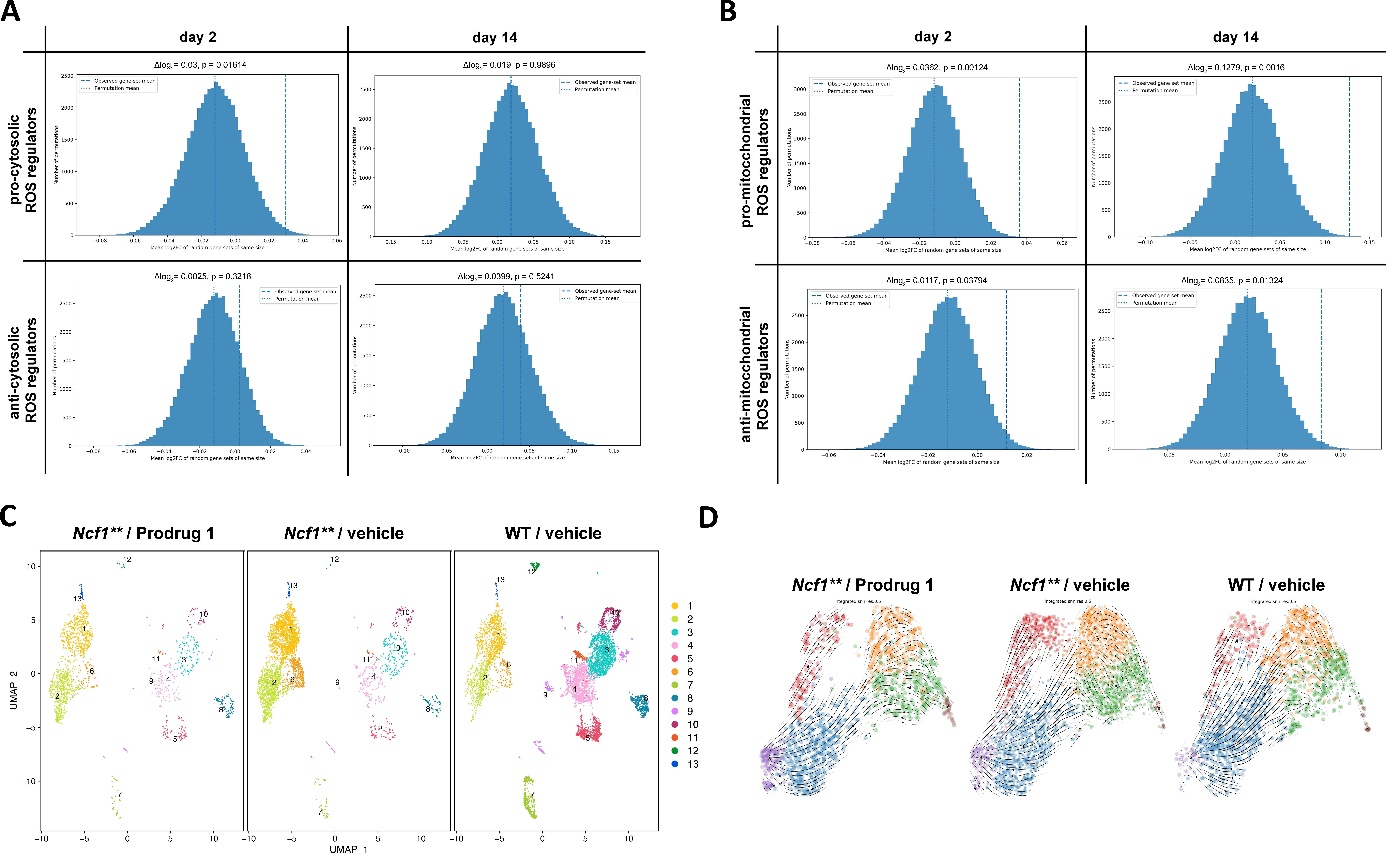


**Figure S5. (A, B)** Permutation analysis of cytosolic (A) and mitochondrial (B) ROS regulators. For each gene, condition-associated expression change was calculated as the mean expression in prodrug **1**-treated samples minus the mean expression in vehicle-treated samples. Positive Δlog_2_ values thus indicate higher expression upon prodrug **1** treatment, negative Δlog_2_ values higher expression under vehicle-treatment. **(C, D)** Single cell analysis of paws 5 days after injection of MSU crystals. **(C)** Non-linear dimensional reduction Uniform Manifold Approximation and Projection (UMAP) plots of all clusters split by different condition. **(D)** RNA velocities of neutrophil cells, computed using the stochastic model with scVelo, are visualized as streamlines on a UMAP embedding for the three conditions.


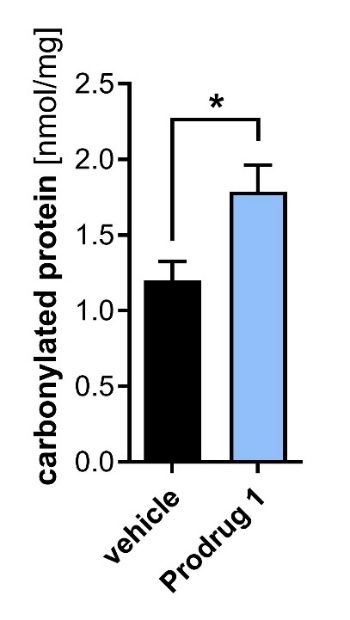


**Figure S6.** Serum concentrations of carbonylated proteins in MSU crystal-injected *Ncf1*** mice after treatment with vehicle (intralipid) or prodrug **1**. N = sera from 4-5 mic per group. *p < 0.05, unpaired Student´s t-test.
